# Supplementary material for: MYC functions as a switch for natural killer cell-mediated immune surveillance of lymphoid malignancies
Source: Nat Commun. 2020 Jun 5;11:2860. doi: 10.1038/s41467-020-16447-7 (PMC7275060; doi:10.1038/s41467-020-16447-7)
Supplement: Supplementary file 1 — Supplementary Information [file 41467_2020_16447_MOESM1_ESM.pdf]

Supplementary Materials (Swaminathan *et al.*)

MYC Functions as a Switch for Natural Killer Cell-Mediated Immune Surveillance of Lymphoid Malignancies

Inventory

- Supplementary Figures S1-S31
- Supplementary Tables S1-S6

Supplementary Figure 1: *MYC-driven lymphomas exhibit disrupted splenic architectures*

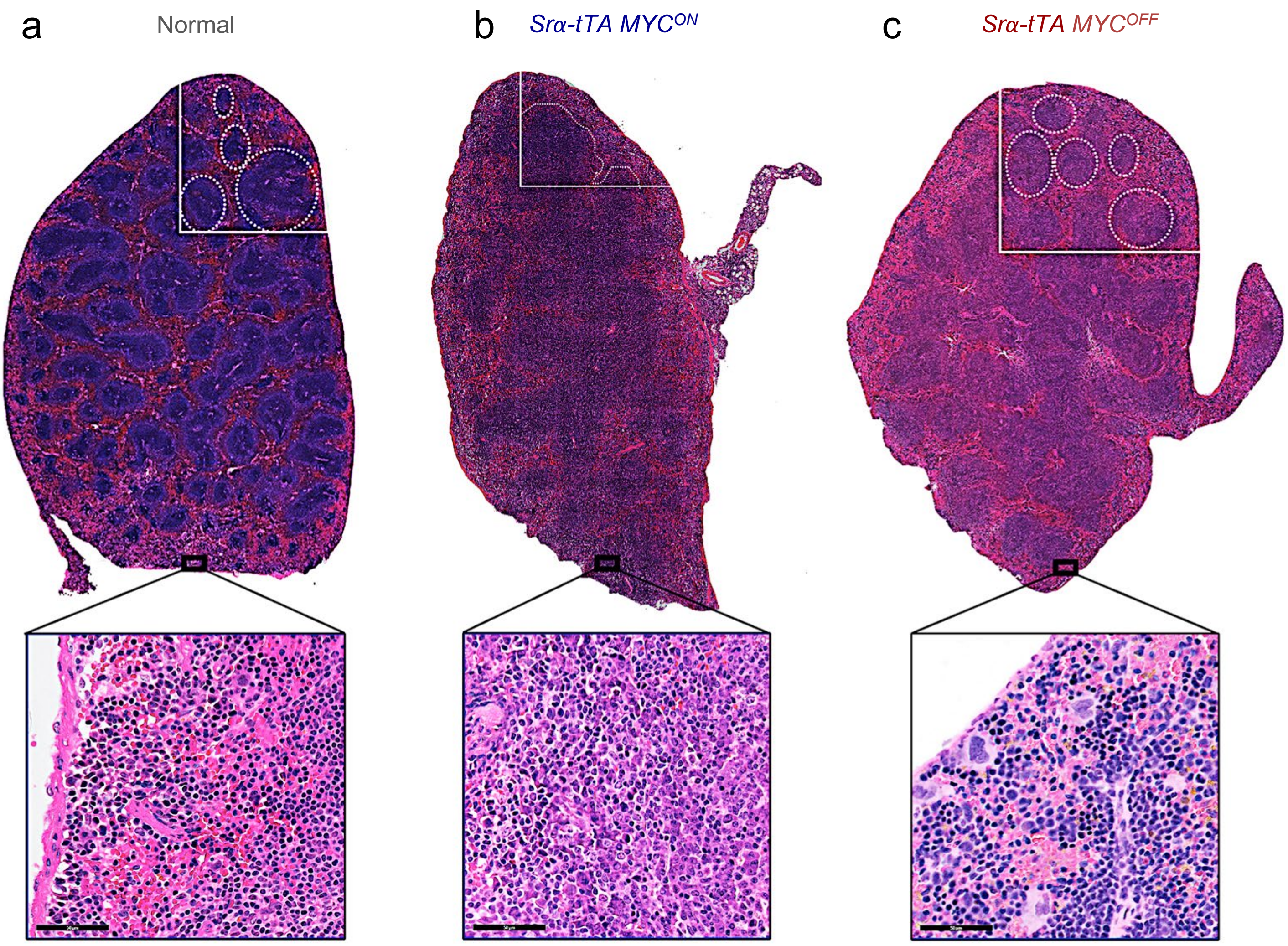

Supplementary Figure 1: MYC-driven lymphomas exhibit disrupted splenic architectures. Hematoxylin and Eosin (H&E) staining of spleens isolated from age and sex matched healthy normal (n = 3, **a**), *Srα-tTA MYC*^{ON} (n = 3, **b**) and *Srα-tTA MYC*^{OFF} (doxycycline 96 h, n = 3, **c**) mice. White dotted circles depict an intact germinal center in healthy and *Srα-tTA MYC*^{OFF} mice. Magnification (top) = 4X, Inset (bottom): Magnification = 20X, Scale bars = 50 μm.

Supplementary Figure 2: *Oncogenic MYC perturbs the relative compositions of splenic immune subsets*

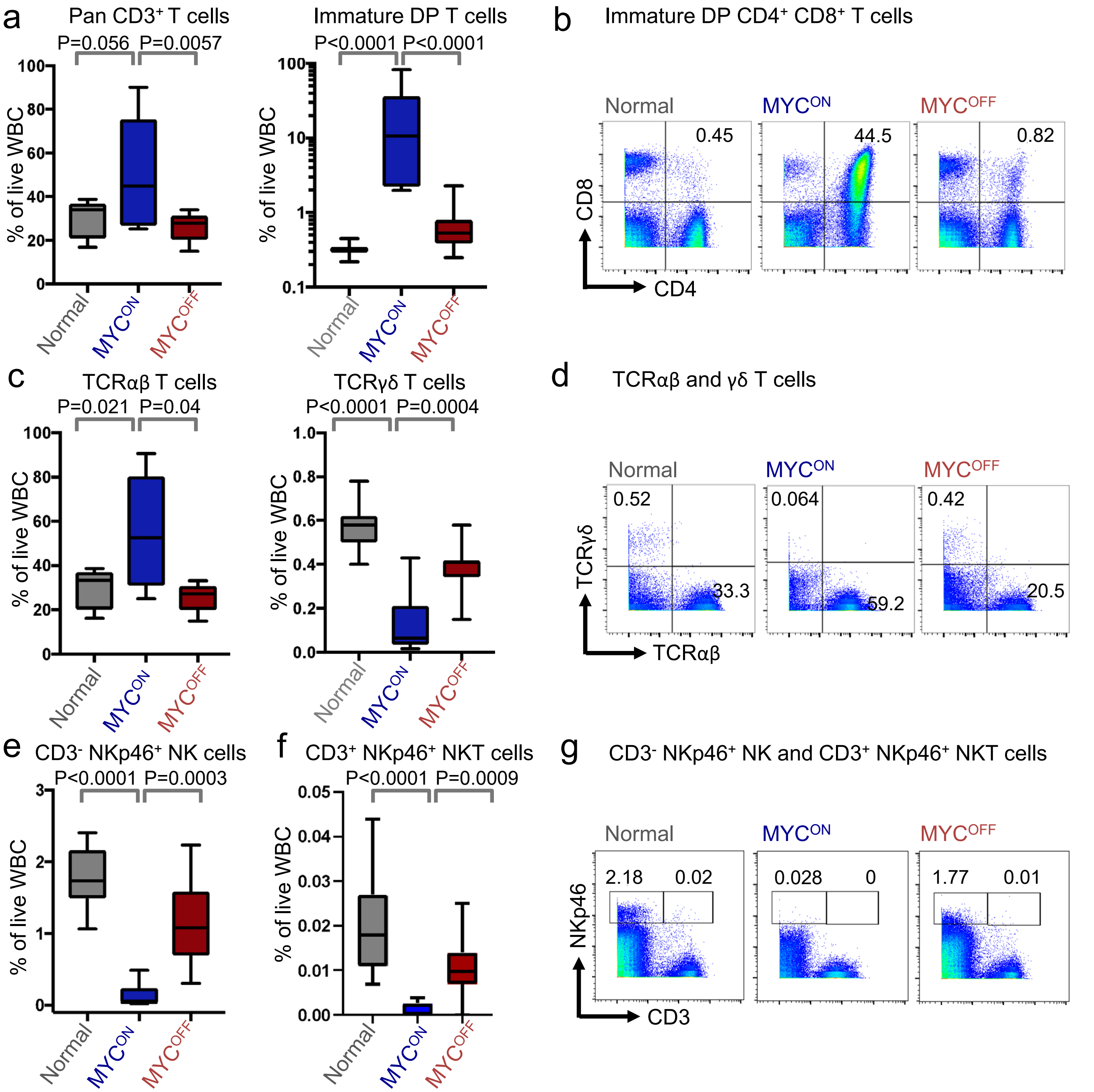

Supplementary Figure 2: Oncogenic MYC perturbs the relative compositions of splenic immune subsets. (**a**) Quantification of CD3$^+$ T and CD4$^+$ CD8$^+$ immature DP T cell percentages in normal (n = 11), *SRα-tTA MYC^{ON}* (n = 9), and *SRα-tTA MYC^{OFF}* (doxycycline 96 h, n = 10) mice by CyTOF. (**b**) Representative CyTOF dot plots showing percentages of DP T cells in spleen from normal, *SRα-tTA MYC^{ON}*, and *SRα-tTA MYC^{OFF}* mice. (**c**) Quantification of TCRαβ$^+$ and TCRγδ$^+$ T cell percentages in normal, *SRα-tTA MYC^{ON}*, and *SRα-tTA MYC^{OFF}* mice. (**d**) Representative CyTOF dot plots showing percentages of TCRαβ and TCRγδ T cells in spleen from normal, *SRα-tTA MYC^{ON}*, and *SRα-tTA MYC^{OFF}* mice. (**e**) Quantification of NK cell percentages in normal, *SRα-tTA MYC^{ON}*, and *SRα-tTA MYC^{OFF}* mice. (**f**) Quantification of NKT cell percentages in normal, *SRα-tTA MYC^{ON}*, and *SRα-tTA MYC^{OFF}* mice. (**g**) Representative CyTOF dot plots showing NK and NKT cell percentages in spleen from normal, *SRα-tTA MYC^{ON}*, and *SRα-tTA MYC^{OFF}* mice. Two-sided p values were calculated using the Mann-Whitney test. For all box plots, center is at the median, minima and maxima are indicated by whiskers, and the box represents data between the 25th and 75th percentile.

Supplementary Figure 3: *Oncogenic MYC perturbs the relative compositions of splenic immune subsets*

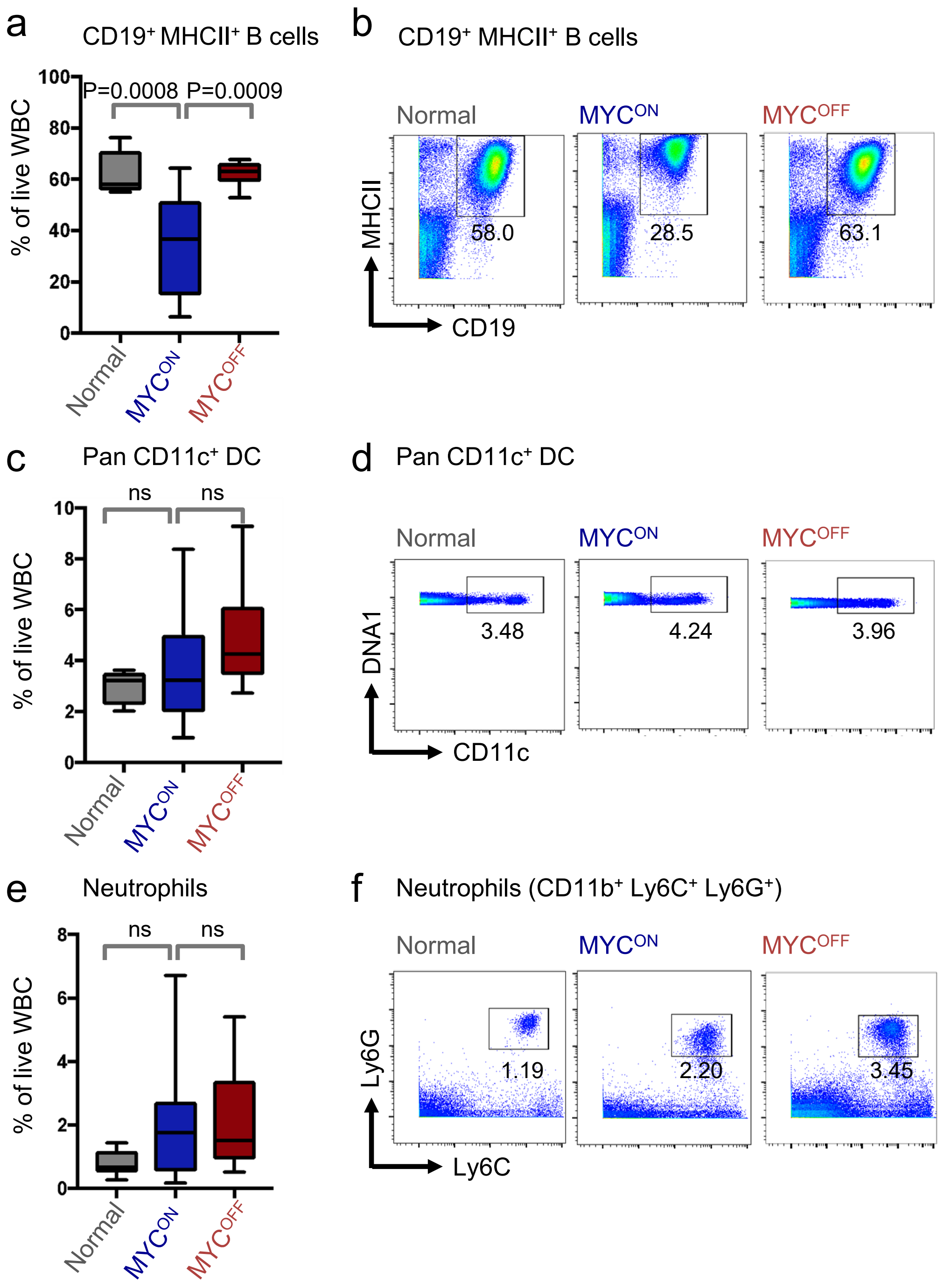

Supplementary Figure 3: Oncogenic MYC perturbs the relative compositions of splenic immune subsets. (**a**) Quantification of CD11c$^+$ DCs in normal (n = 11), *SRα-tTA MYC^{ON}* (n = 9), and *SRα-tTA MYC^{OFF}* (doxycycline 96 h, n = 10) mice by CyTOF. (**b**) CyTOF dot plots showing percentages of DCs in one representative spleen from normal, *SRα-tTA MYC^{ON}*, and *SRα-tTA MYC^{OFF}* mice. (**c**) Quantification of neutrophil percentages in normal, *SRα-tTA MYC^{ON}*, and *SRα-tTA MYC^{OFF}* mice. (**d**) CyTOF dot plots showing percentages of neutrophils in one representative spleen from normal, *SRα-tTA MYC^{ON}*, and *SRα-tTA MYC^{OFF}* mice. (**e**) Quantification of B cell percentages in normal, *SRα-tTA MYC^{ON}*, and *SRα-tTA MYC^{OFF}* mice. (**f**) CyTOF dot plots showing B cell percentages in one representative spleen from normal, *SRα-tTA MYC^{ON}*, and *SRα-tTA MYC^{OFF}* mice. All populations have been gated on live intact singlets. Two-sided p values were calculated using the Mann-Whitney test (ns = not significant). For all box plots, center is at the median, minima and maxima are indicated by whiskers, and the box represents data between the 25th and 75th percentile.

Supplementary Figure 4: *Changes in absolute numbers of splenic immune subsets during MYC-driven lymphomagenesis*

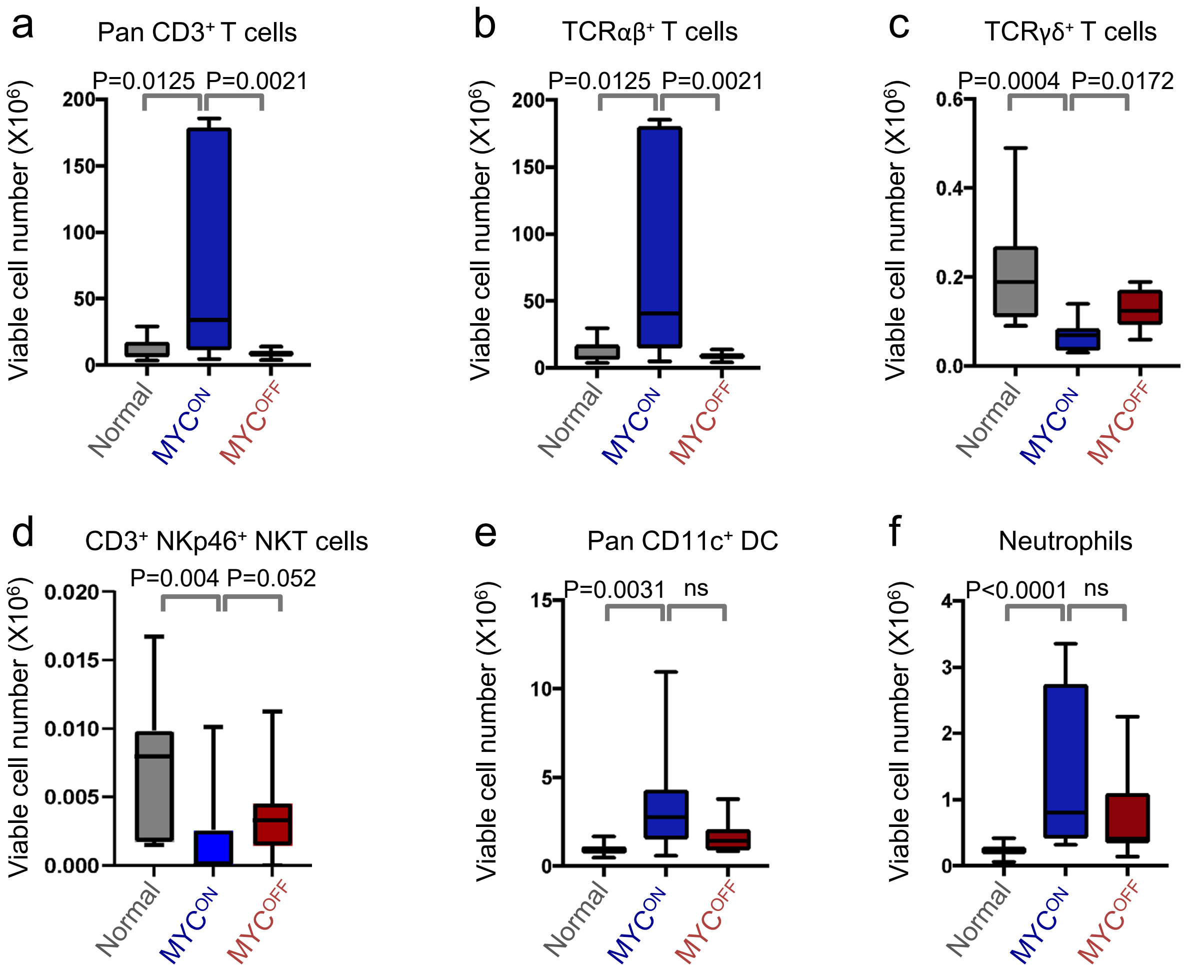

Supplementary Figure 4: Changes in absolute numbers of splenic immune subsets during MYC-driven lymphomagenesis. (a-f) Quantification of absolute cell numbers of pan $CD3^{+}$ T cells (a), $TCR\alpha\beta^{+}$ T cells (b), $TCR\gamma\delta^{+}$ T cells (c), $CD3^{+}$ $NKp46^{+}$ NKT cells (d), pan $CD11c^{+}$ DC (e), and neutrophils (f), in spleens of normal (n = 11), *SRα-tTA* MYC^{ON} (n = 9), and *SRα-tTA* MYC^{OFF} (doxycycline 96 h, n = 10) mice subjected to mass cytometry. Two-sided p values were calculated using the Mann-Whitney test (ns = not significant). For all box plots, center is at the median, minima and maxima are indicated by whiskers, and the box represents data between the 25th and 75th percentile.

Supplementary Figure 5: *Alterations of splenic NK compositions in MYC-driven T cell lymphoma verified by flow cytometry*

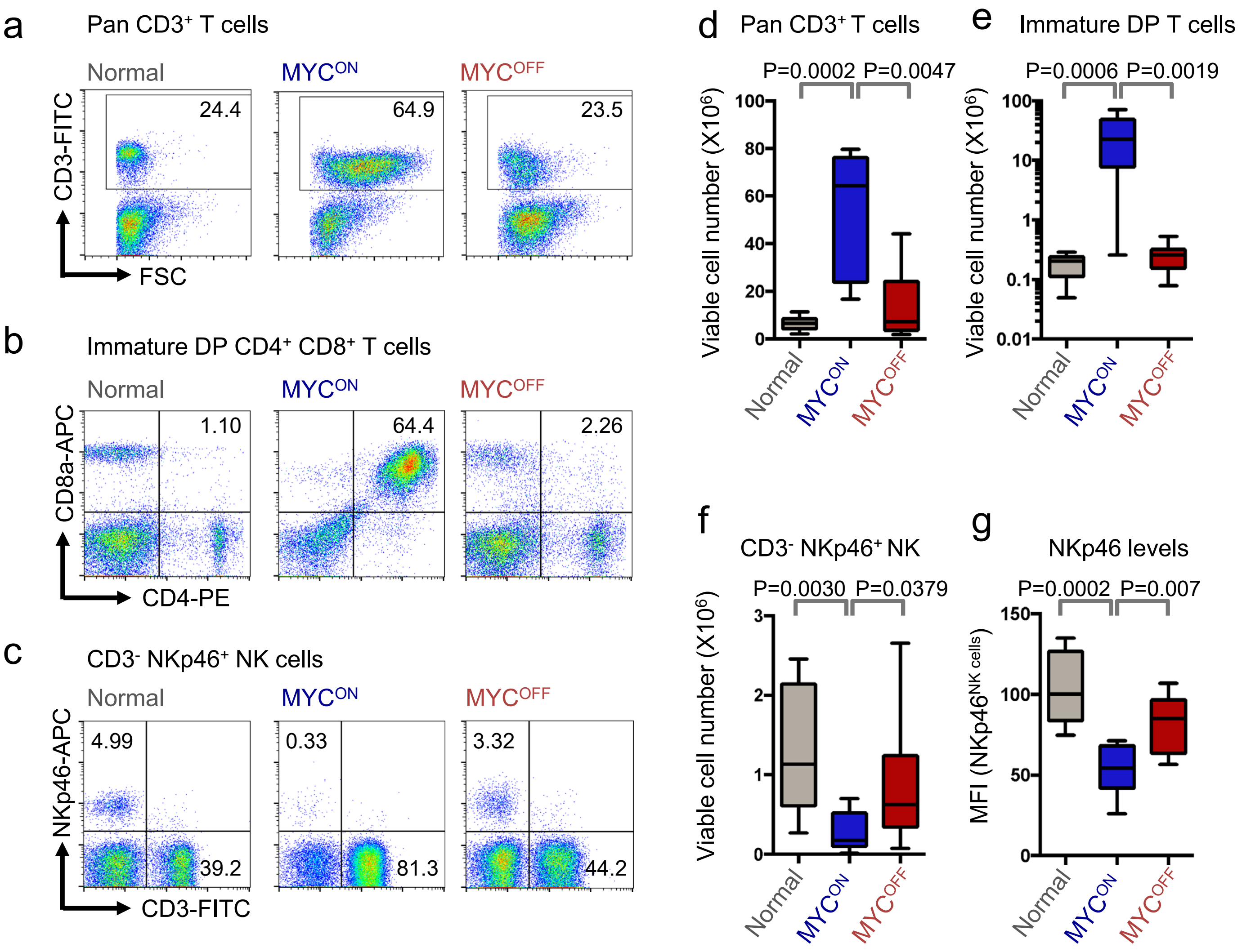

Supplementary Figure 5: Alterations of splenic NK compositions in MYC-driven T cell lymphoma. (**a-c**) Representative flow cytometry plots depicting splenic pan $CD3^+$ T cell (a), $CD4^+$ $CD8^+$ DP T cells (b), and $CD3^-$ $NKp46^+$ NK cell (c), distributions in normal (n = 8), *SRα-tTA* MYC^{ON} (n = 8), and *SRα-tTA* MYC^{OFF} (doxycycline 96 h, n = 8) mice. (**d-f**) Quantification of numbers of pan $CD3^+$ T cells (d), and immature DP T cell (e), and $CD3^-$ $NKp46^+$ NK cells (f) in normal (n = 8), *SRα-tTA* MYC^{ON} (n = 8), and *SRα-tTA* MYC^{OFF} (n = 8) spleens by flow cytometry. (**g**) MFI of surface NKp46 in splenic NK cells from normal (n = 8), *SRα-tTA* MYC^{ON} (n = 8), and *SRα-tTA* MYC^{OFF} (n = 8) mice. All populations are depicted as percentages of live cells (FSC^+ PI^-). Two-sided p values were calculated using the Mann-Whitney test (ns = not significant). For all box plots, center is at the median, minima and maxima are indicated by whiskers, and the box represents data between the 25th and 75th percentile.

Supplementary Figure 6: *CIBERSORT measuring changes in splenic NK composition during murine MYC-driven T cell lymphomagenesis*

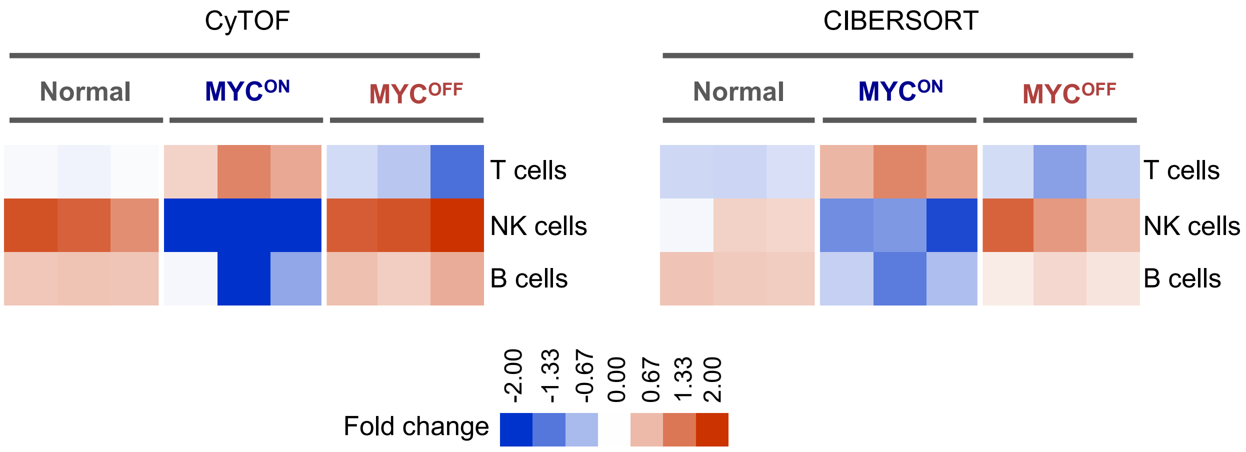

Supplementary Figure 6: CIBERSORT measuring changes in splenic NK composition during murine MYC-driven T cell lymphomagenesis. Heatmap comparing fold changes in T, B and NK immune subsets in normal (n = 3), *SRα-tTA MYC*^{ON} (n = 3), and *SRα-tTA MYC*^{OFF} (n = 3) mice by CyTOF (left) and CIBERSORT (right).

Supplementary Figure 7: *Suppression of bone marrow NK cells during MYC-driven lymphomagenesis*

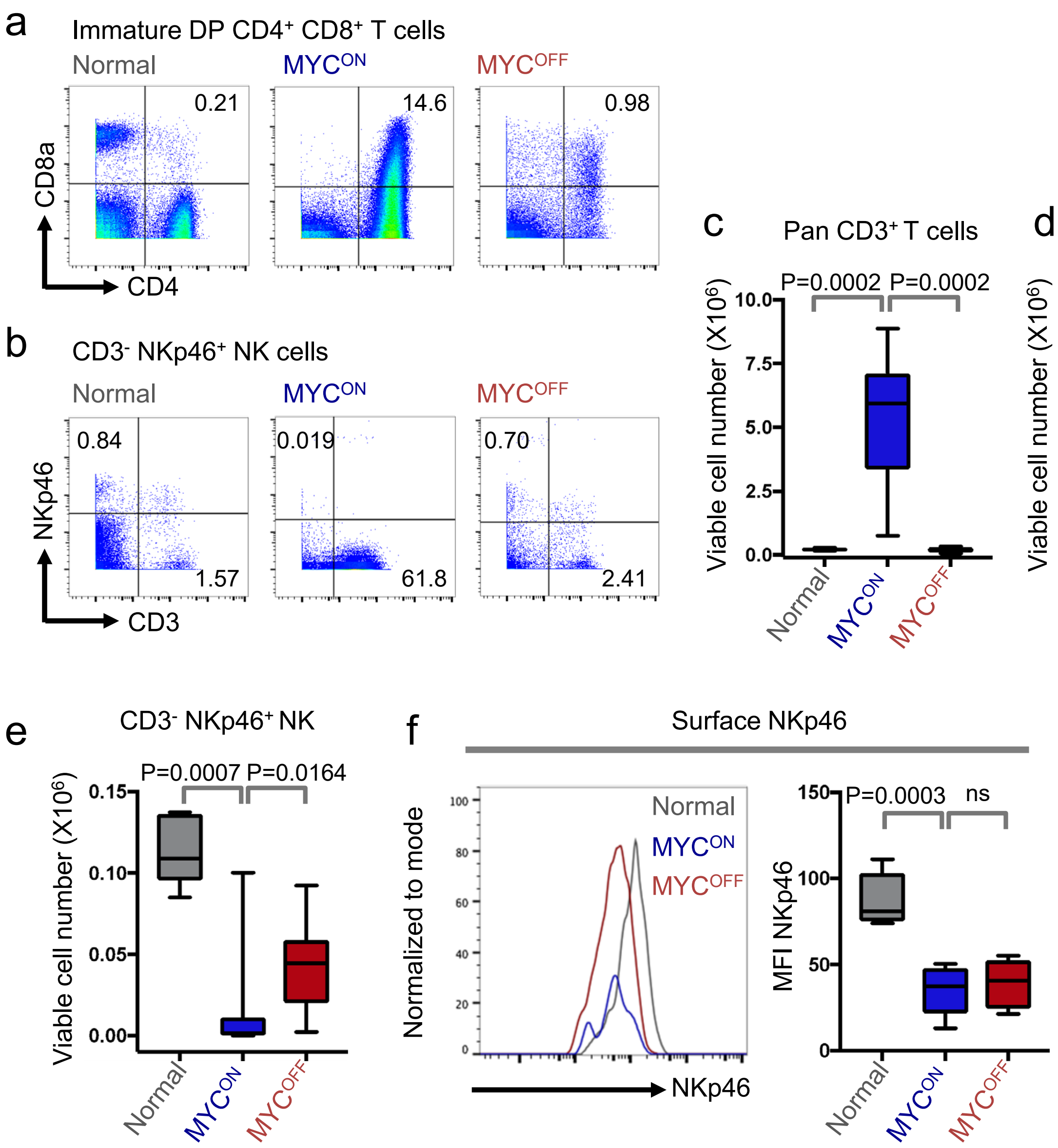

Supplementary Figure 7: Suppression of bone marrow NK cells during MYC-driven lymphomagenesis. (**a-b**) Representative CyTOF plots depicting T and NK cell distributions in normal (n = 7), *SRα-tTA MYC^ON* (n = 9), and *SRα-tTA MYC^OFF* (doxycycline 96 h, n = 7) bone marrows. (**c-e**) Quantification of absolute numbers of pan $CD3^+$ T cells (c), immature DP T cells (d), and NK cells (e) in bone marrows of normal, *SRα-tTA* MYC^{ON}, and *SRα-tTA* MYC^{OFF} mice. (**f**) Representative MFI of surface NKp46 in bone marrow NK cells from normal, *SRα-tTA* MYC^{ON}, and *SRα-tTA* MYC^{OFF} mice. All populations are depicted as percentages of live intact singlets. Two-sided p values were calculated using the Mann-Whitney test (ns = not significant). For all box plots, center is at the median, minima and maxima are indicated by whiskers, and the box represents data between the 25th and 75th percentile.

Supplementary Figure 8: *Increased cell death is not responsible for reduction of NK cells in MYC-driven lymphomas*

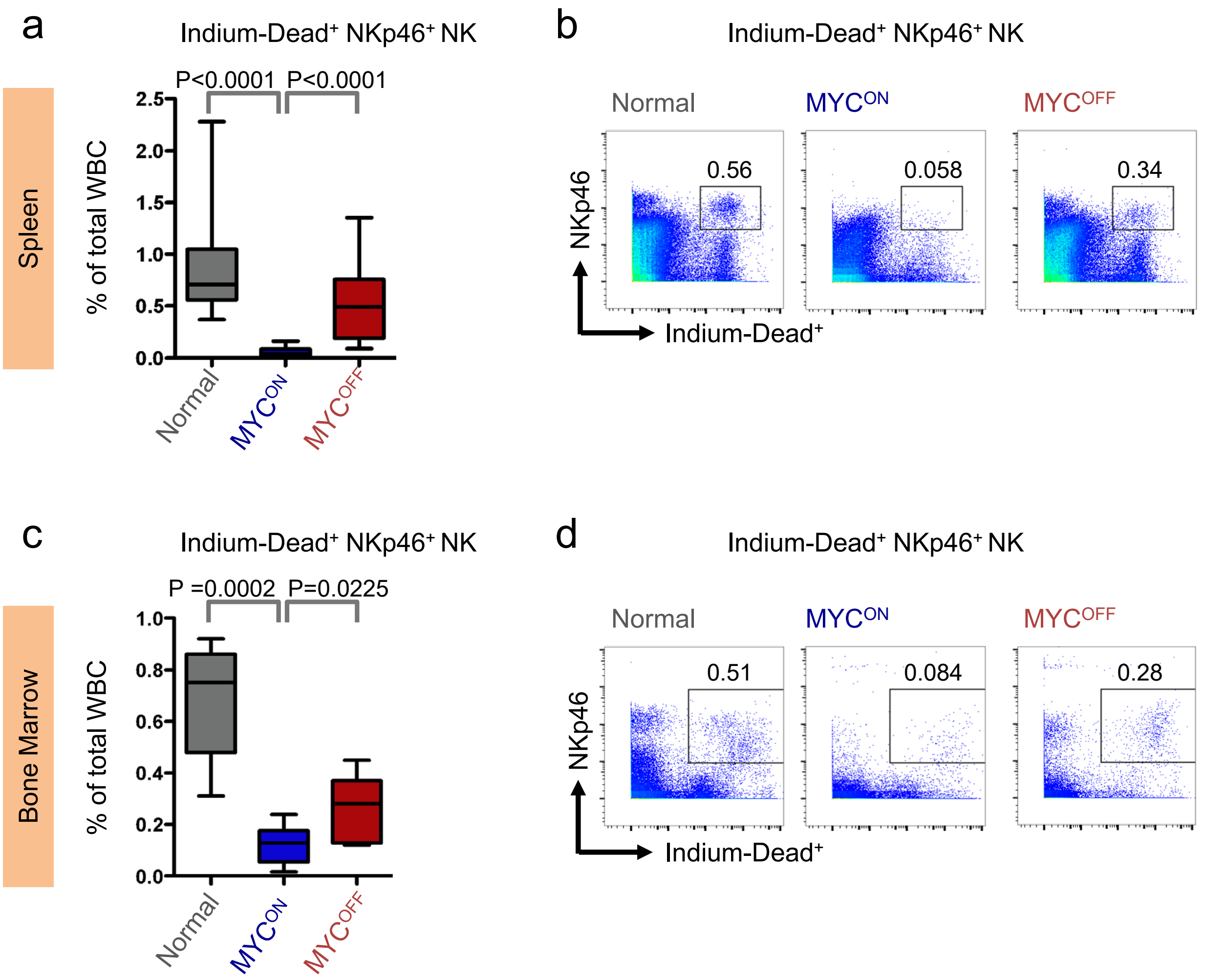

Supplementary Figure 8: Increased cell death is not responsible for reduction of NK cells in MYC-driven lymphomas. (**a-b**) Quantification of percentages (a), and representative plots (b) of dead NK cells (Indium-Dead$^+$ NKp46$^+$) in normal (n = 11), *SRα-tTA MYC^{ON}* (n = 9), and *SRα-tTA MYC^{OFF}* (doxycycline 96 h, n = 10) spleens measured by CyTOF. (**c-d**) Quantification of percentages (c), and representative plots (d) of dead NK cells (Indium-Dead$^+$ NKp46$^+$) in normal (n = 7), *SRα-tTA MYC^{ON}* (n = 9), and *SRα-tTA MYC^{OFF}* (doxycycline 96 h, n = 7) bone marrows measured by CyTOF. Two-sided p values were calculated using the Mann-Whitney test (ns = not significant). For all box plots, center is at the median, minima and maxima are indicated by whiskers, and the box represents data between the 25th and 75th percentile.

Supplementary Figure 9: *Strain-specific expression pattern of CD27 and CD11b on mature NK cells*

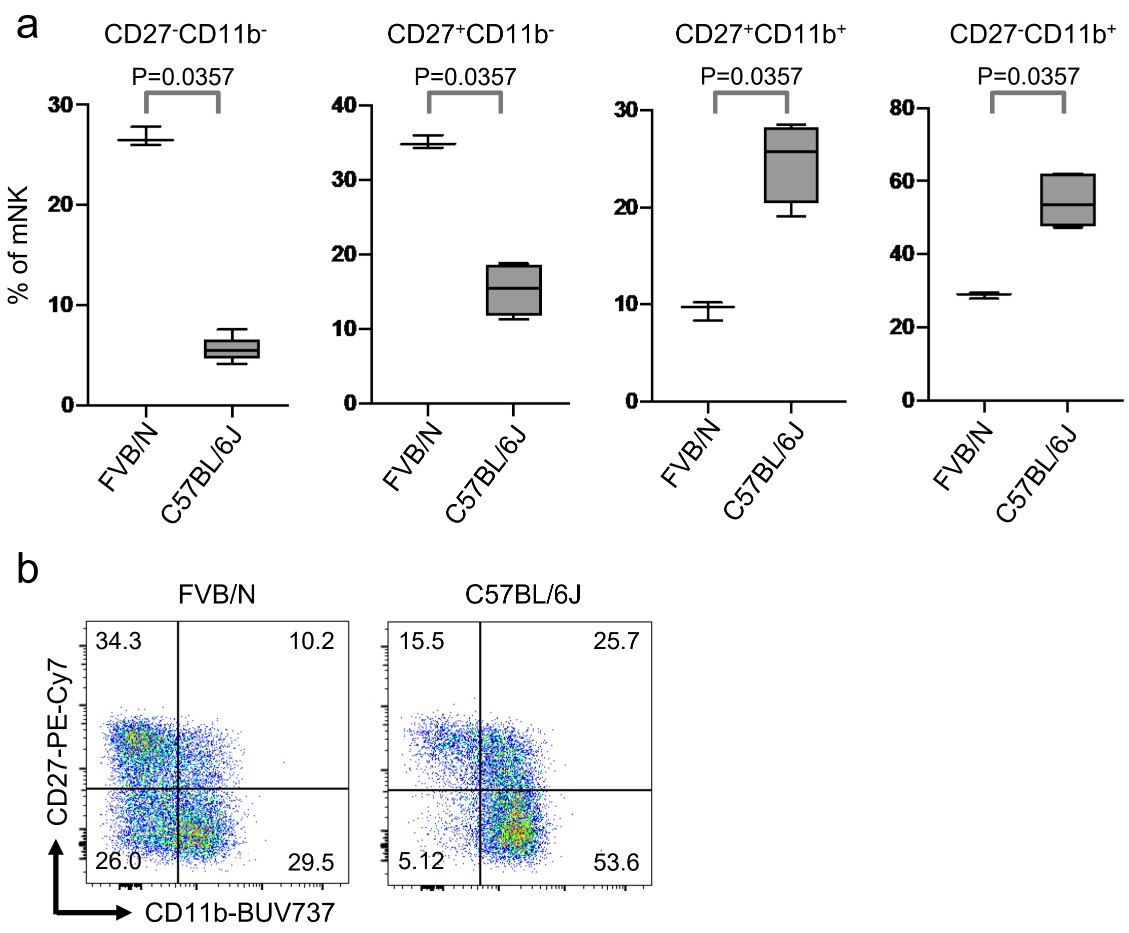

Supplementary Figure 9. Strain-specific expression pattern of CD27 and CD11b on mature NK cells. (a-b) Quantification of percentages (a) and representative flow plots (b) of mNK cells expressing CD27 and CD11b in spleen in FVB/N wt (n = 3) and C57BL/6J wt (n = 5) mice. Populations were gated on $Lin^{-}CD122^{+}NKp46^{+}CD49b^{+}$ mature NK (mNK) intact live singlets. Two-sided p values were calculated using the Mann-Whitney test. For all box plots, center is at the median, minima and maxima are indicated by whiskers, and the box represents data between the 25th and 75th percentile.

Supplementary Figure 10: *Doxycycline does not impact NK cell maturation in FVB/N wt*

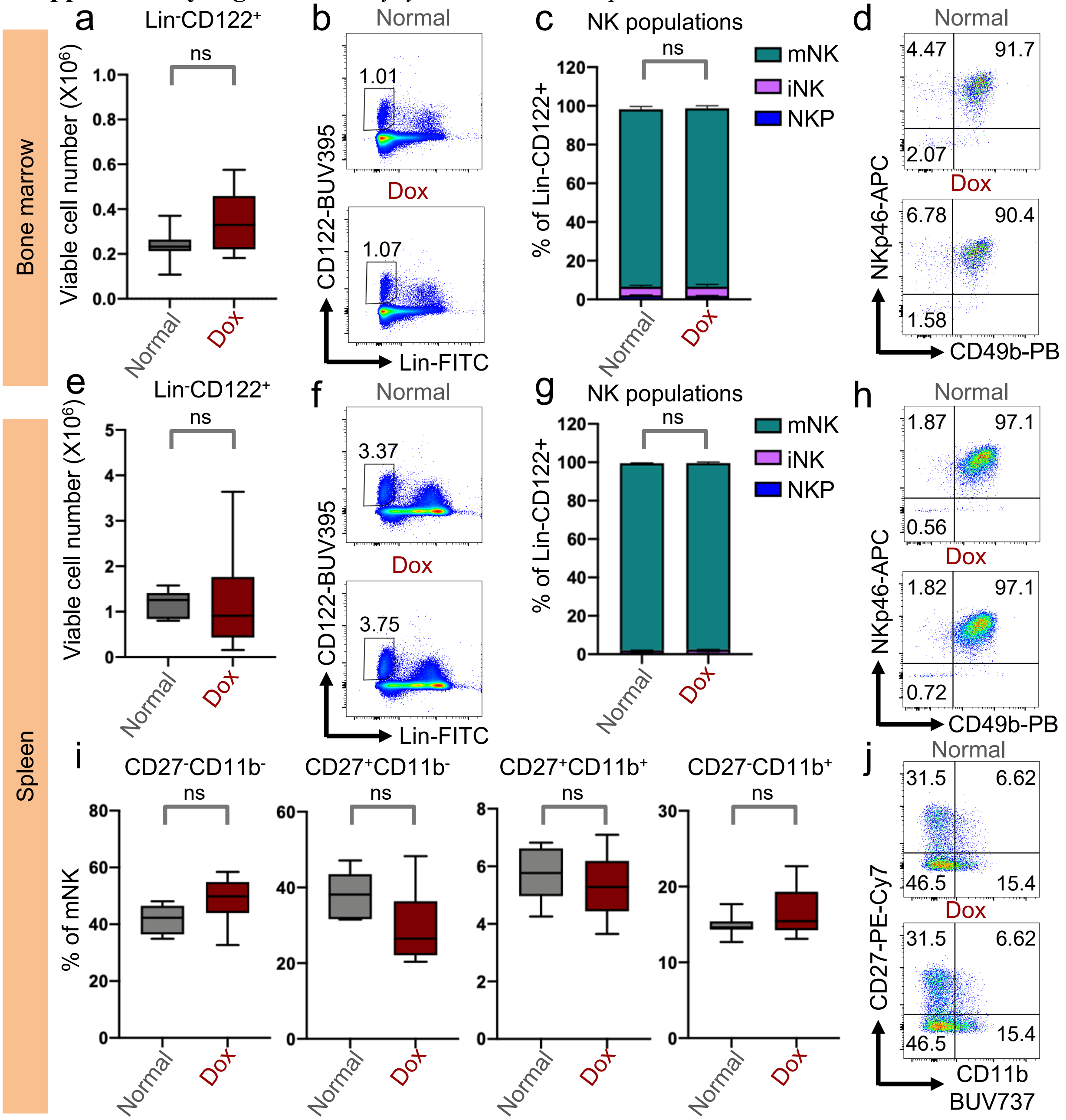

Supplementary Figure 10. Doxycycline does not impact NK cell maturation in FVB/N wt. FVB/N wt mice were left untreated (Normal) or treated with doxycycline for 96 hrs (Dox). (**a-b**) Quantification of absolute cell number of Lin⁻CD122⁺ cells (a) and representative flow plots (b) in bone marrow measured by flow cytometry of Normal (n = 7) and Dox treated mice (n = 6). (**c-d**) Quantification of percentages (c) and representative flow plots (d) of NKP, iNK and mNK from bone marrow in Normal (n = 7) and Dox treated mice (n = 6). Data in (c) show mean+SD. (**e-f**) Quantification of absolute cell number of Lin⁻CD122⁺ cells (e) and representative flow plots (f) in spleen measured by flow cytometry of Normal (n = 7) and Dox treated mice (n = 6). (**g-h**) Quantification of percentages (g) and representative flow plots (h) of NKP, iNK and from spleen of Normal (n = 7) and Dox treated mice (n = 6). Data in (g) show mean+SD (**i-j**) Quantification of percentages (i) and representative flow plots (j) of mNK cells expressing CD27 and CD11b in spleen measured by flow cytometry of Normal (n = 7) and Dox treated mice (n = 6). Two-sided p values were calculated using the Mann-Whitney test (ns = not significant). For all box plots, center is at the median, minima and maxima are indicated by whiskers, and the box represents data between the 25th and 75th percentile. Lin = Lineage.

Supplementary Figure 11: *Peripheral involvement of MYC-driven lymphomagenesis*

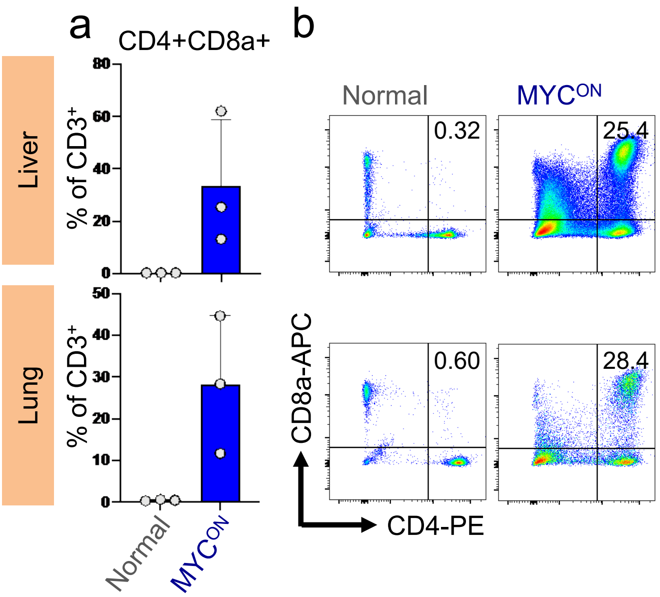

Supplementary Figure 11. Peripheral involvement of MYC-driven lymphomagenesis. (a-b) Quantifications of percentages (a) and representative flow plots (b) of $CD4^{+}CD8^{+}$ double positive lymphoblasts gated on $CD3^{+}$ live intact singlets from liver and lung in normal (n = 3) and *SRα-tTA MYC^{ON}* (n = 3). The bars in (a) indicate mean+SD.

Supplementary Figure 12: *MYC driven lymphomagenesis prevents NK maturation in liver and lung.*

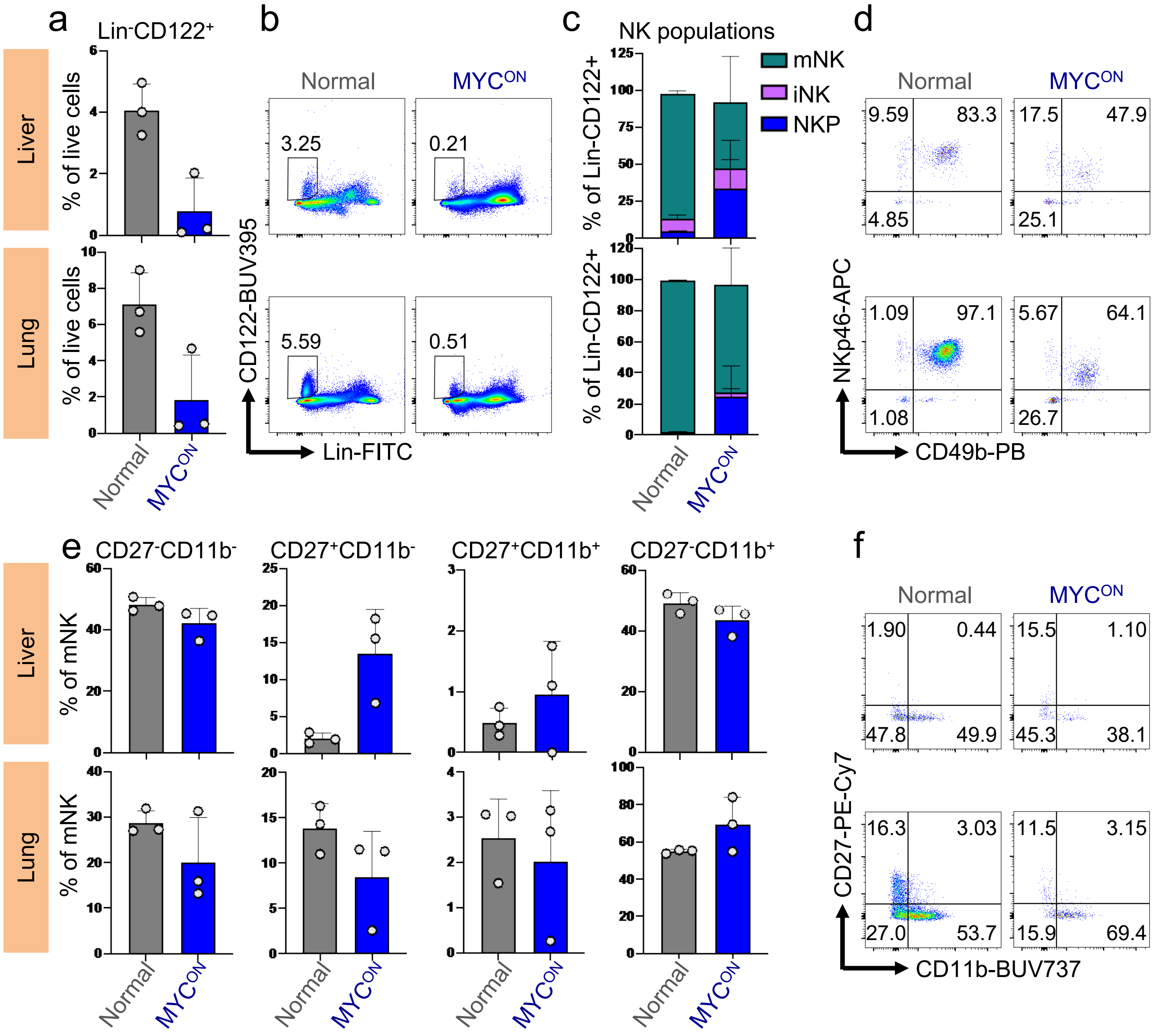

Supplementary Figure 12. MYC driven lymphomagenesis prevents NK maturation in liver and lung. (**a-b**) Quantification of percentages of Lin$^-$CD122$^+$ cells (a) and representative flow plots (b) in liver and lung in normal (n = 3) and *SRα-tTA MYC^{ON}* (n = 3) mice measured by flow cytometry. The bars in (a) indicate mean+SD. (**c-d**) Quantification of percentages (c) and representative flow plots (d) of NKP, iNK and mNK gated on the Lin$^-$CD122$^+$ live intact singlets from liver and lung in normal (n = 3) and *SRα-tTA MYC^{ON}* (n = 3). Data in (c) show mean+SD. (**e-f**) Quantification of percentages (e) and representative flow plots (f) of mNK cells expressing CD27 and CD11b in liver and lung in normal (n = 3) and *SRα-tTA MYC^{ON}* (n = 3) mice. Populations are gated on Lin$^-$CD122$^+$NKp46$^+$CD49b$^+$ intact live singlets. The bars in (e) indicate mean+SD.

Supplementary Figure 13: *Transcriptional repression of key NK cell genes during primary MYC-driven T cell lymphomagenesis*

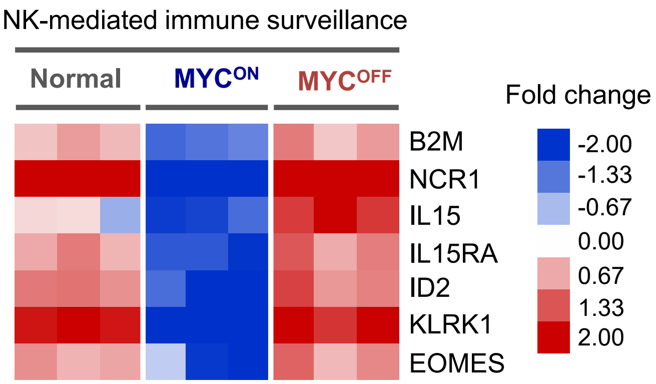

Supplementary Figure 13. Transcriptional repression of key NK cell genes during primary MYC-driven T cell lymphomagenesis. Heatmap comparing expression of key NK cell genes in splenic samples from normal (n = 3), *SRα-tTA MYC*^{ON} (n = 3), and *SRα-tTA MYC*^{OFF} (n = 3) mice.

Supplementary Figure 14: *Transcriptional repression of Type I IFN signaling during murine MYC-driven B cell lymphomagenesis*

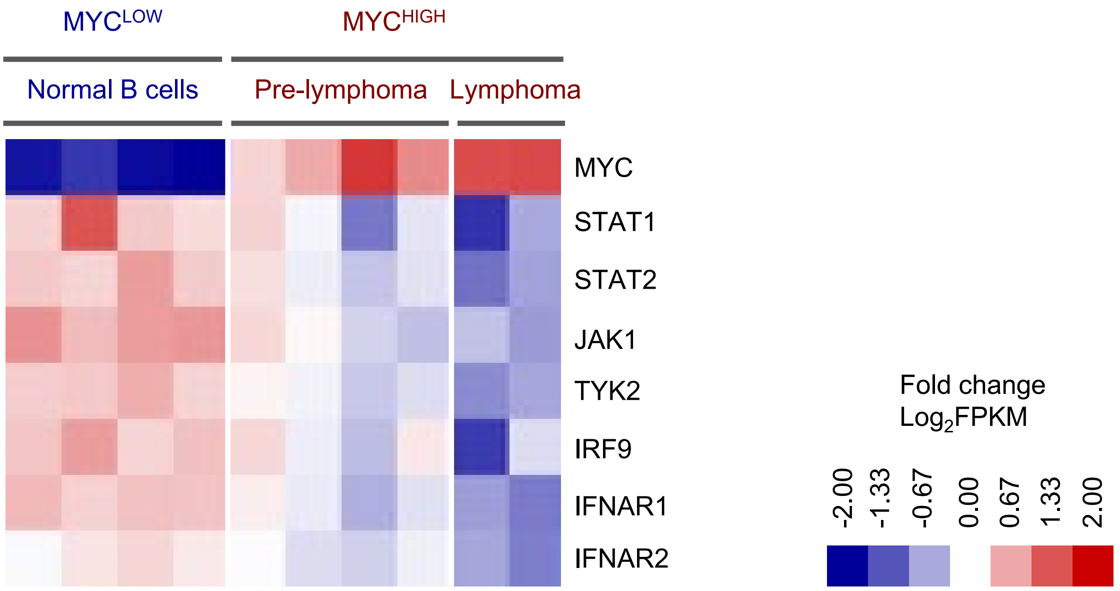

Supplementary Figure 14: Transcriptional repression of Type I IFN signaling during murine MYC-driven B cell lymphomagenesis. Heat map comparing transcriptional changes in Type I IFN signaling genes in normal B cells from healthy mice (MYC^{LOW}, n = 4), pre-lymphomagenic B cells from *Eμ-MYC* mice (pre-lymphoma, MYC^{HIGH}, n = 4), and in full blown primary B cell lymphomas from *Eμ-MYC* mice (MYC^{HIGH}, n = 2) using RNA sequencing (GSE51008).

Supplementary Figure 15: *Short term IFNα administration does not affect lymphoma T cell count.*

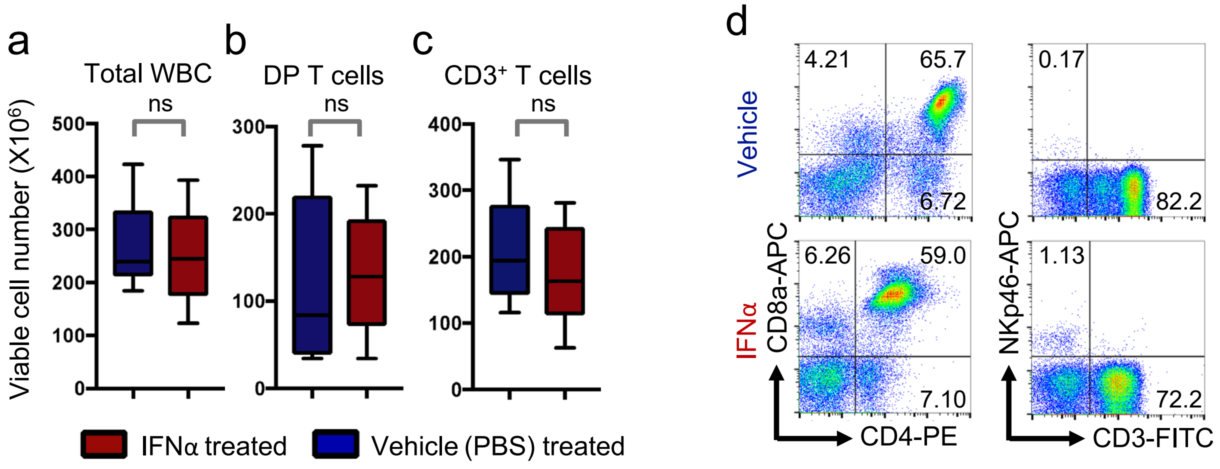

Supplementary Figure 15: Short term IFNa administration does not affect lymphoma T cell count. (a-c) Quantification of absolute counts of total WBC (**a**), $CD4^{+}$ $CD8^{+}$ DP T (**b**) and $CD3^{+}$ pan T (**c**) in *SRα-tTA/tet-O-MYC* mice bearing overt lymphomas (*SRα-tTA* MYC^{ON}) treated for three days with either IFNα (n = 5), or vehicle (PBS, n = 5). (**d**) Representative flow cytometry plots comparing percentages of $CD4^{+}$ $CD8^{+}$ DP T (left) and $CD3^{+}$ $NKp46^{-}$ NK (right) cells between *SRα-tTA/tet-O-MYC* mice in IFNα-treated and vehicle (PBS)-treated groups for three days. Two sided p-values were calculated using the Mann-Whitney test (ns = not significant). For all box plots, center is at the median, minima and maxima are indicated by whiskers, and the box represents data between the 25th and 75th percentile.

Supplementary Figure 16: *Blocking IFNAR1 does not impair splenic NK cell replenishment in SRα-tTA MYC^{OFF}*

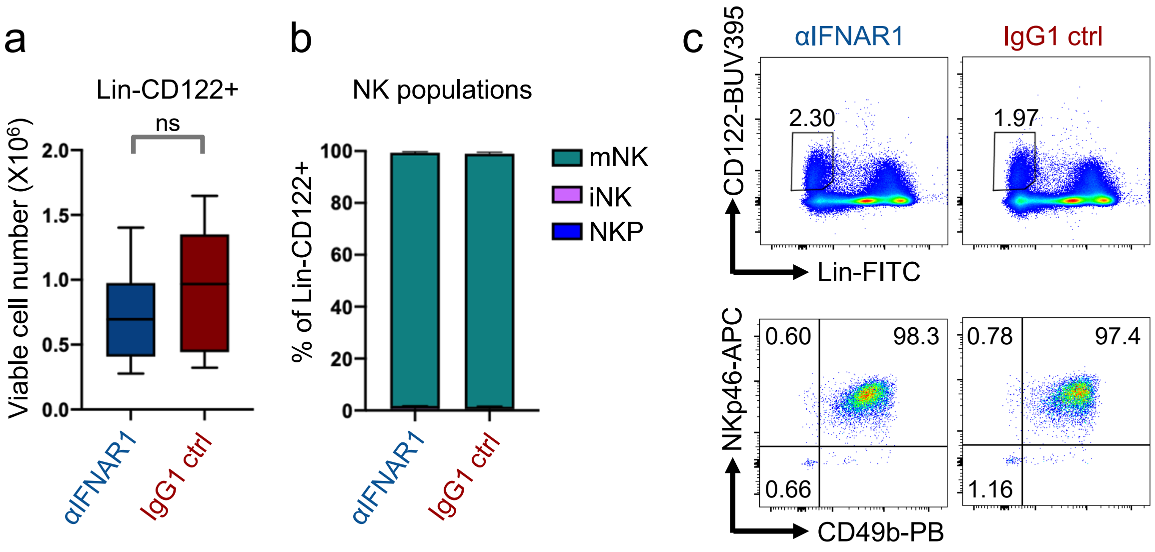

Supplementary Figure 16: Blocking IFNαR1 does not impair splenic NK cell replenishment in SRα-tTA MYC^{OFF} mice. (**a**) Quantification of absolute cell number of Lin$^-$CD122$^+$ in spleen of SRα-tTA MYC^{OFF} (doxycycline 96 h) treated with anti-IFNαR1 (n = 7) or IgG1 control (n = 5) during the four-days doxycycline treatment period. ns = non-significant calculated by Mann-Whitney test. (**b)** Quantification of percentages of NKP, iNK and mNK gated on the Lin$^-$CD122$^+$ live intact singlets from spleen of of *SRα-tTA MYC^{OFF}* (doxycycline 96h) treated with anti-IFNαR1 (n = 7) or IgG1 control (n = 5) during the four-days doxycycline treatment period. Data show mean+SD. (**c**) Representative flow plots for expression of Lineage markers, CD122, NKp46 and CD49b. Two-sided p values were calculated using the Mann-Whitney test (ns = not significant). For all box plots, center is at the median, minima and maxima are indicated by whiskers, and the box represents data between the 25th and 75th percentile.

Supplementary Figure 17: *Signaling and surface receptor changes after MYC inactivation in human BL model*

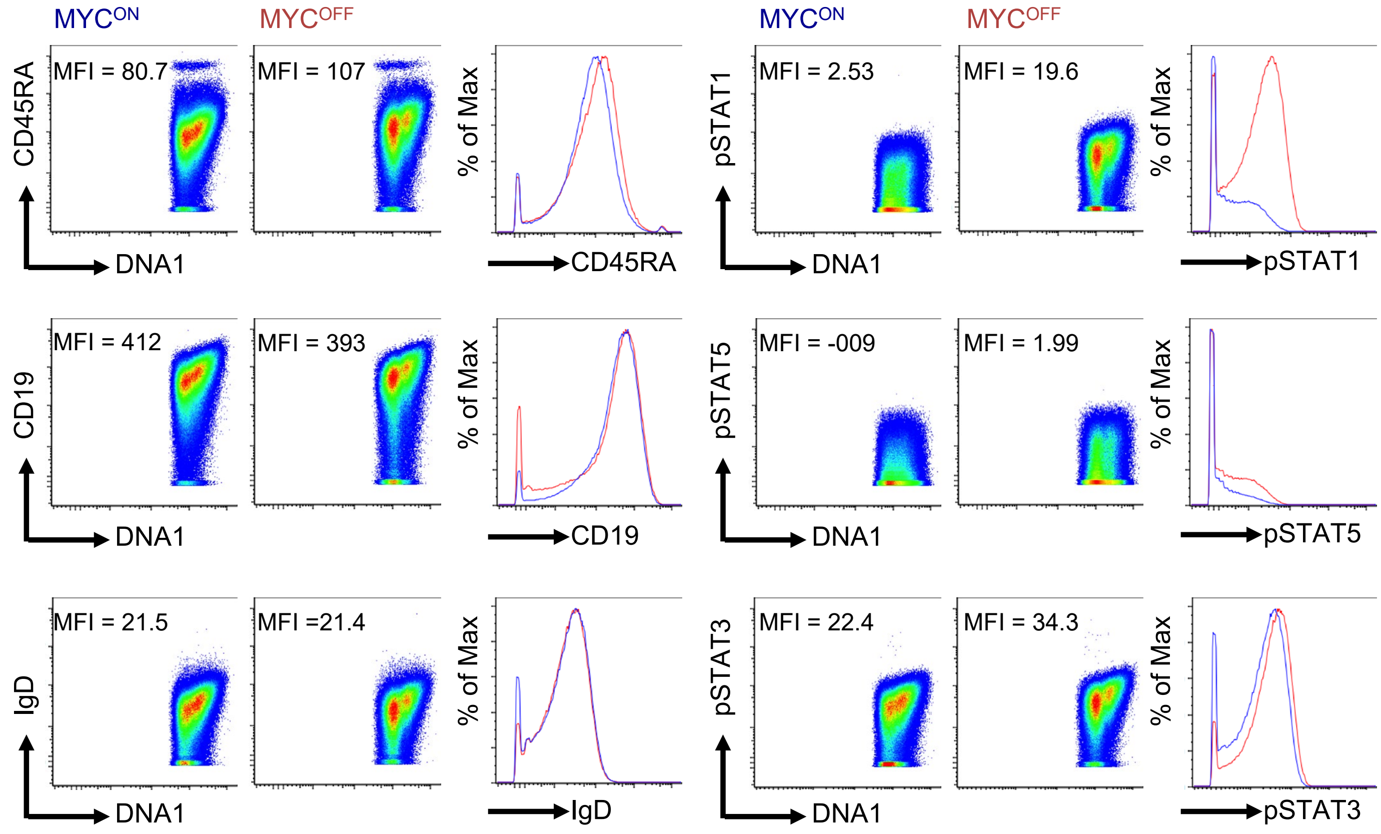

Supplementary Figure 17: Signaling and surface receptor changes after MYC inactivation in human BL model. Dot plots of Phospho-CyTOF depicting the global changes in B cell specific surface receptors and cytokine JAK-STAT signaling pathways after turning MYC off for 24h in P493-6 cells.

Supplementary Figure 18: *Full scan of blot shown in Figure 5c*

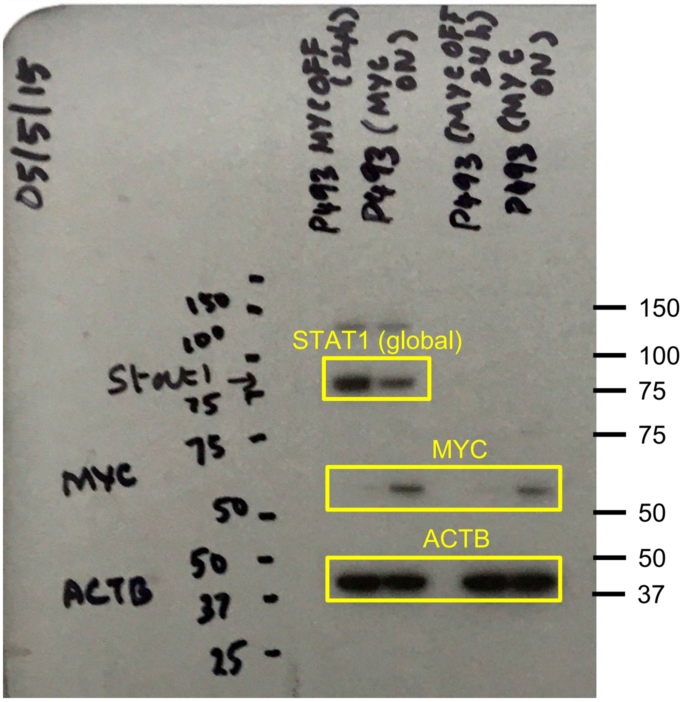

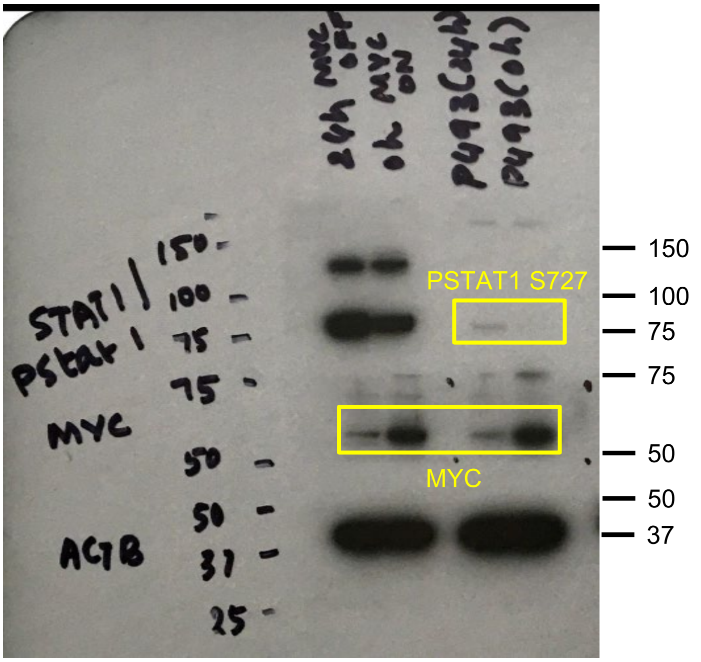

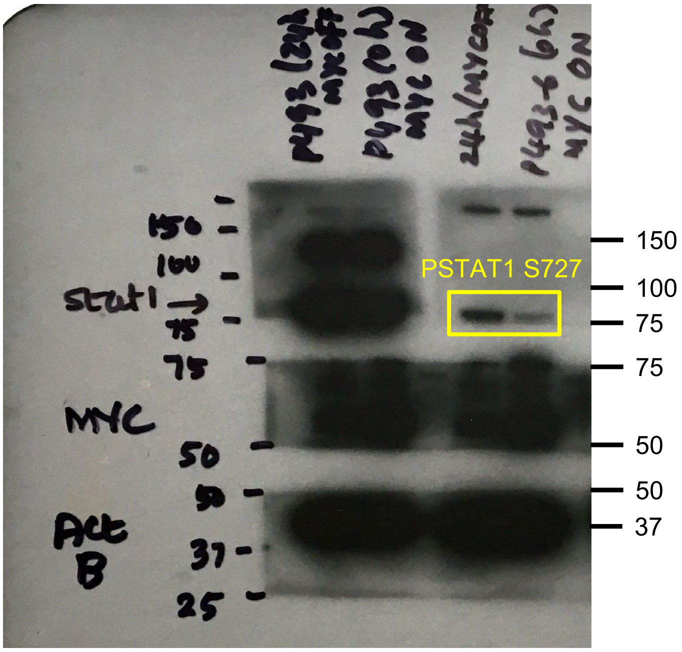

Lanes (left to right):

Ladder
P493-6 MYC OFF 24h
P493-6 MYC ON 0h
Ladder
P493-6 MYC OFF 24h
P493-6 MYC ON 0h
Ladder

Supplementary Figure 18: Full scan of blot shown in Figure 5c. Blot was developed on a film.

Supplementary Figure 19: *High MYC levels in B cell lymphomas block transcription of STAT1 and STAT2*

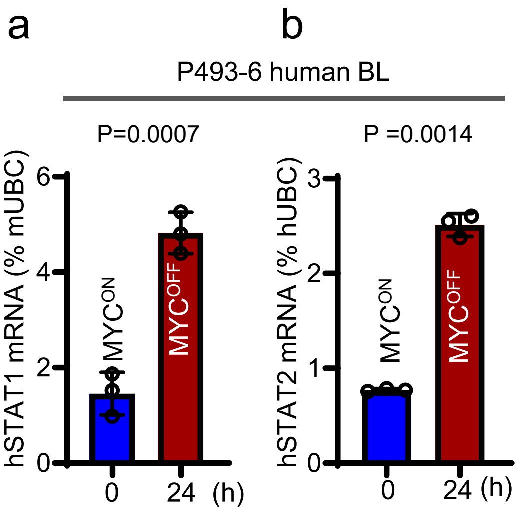

Supplementary Figure 19: High MYC levels in B cell lymphomas block transcription of STAT1 and STAT2. (**a-b**) Quantitative real time PCR for hSTAT1 (a) and, hSTAT2 (b) in P493-6 human BL cell line before and after MYC inactivation by doxycycline for 24 hours (n = 3, mean ± s.d). Two-sided p-values have been calculated using the Student's t-test.

Supplementary Figure 20: *MYC inactivation in BL changes the secreted cytokine profile*

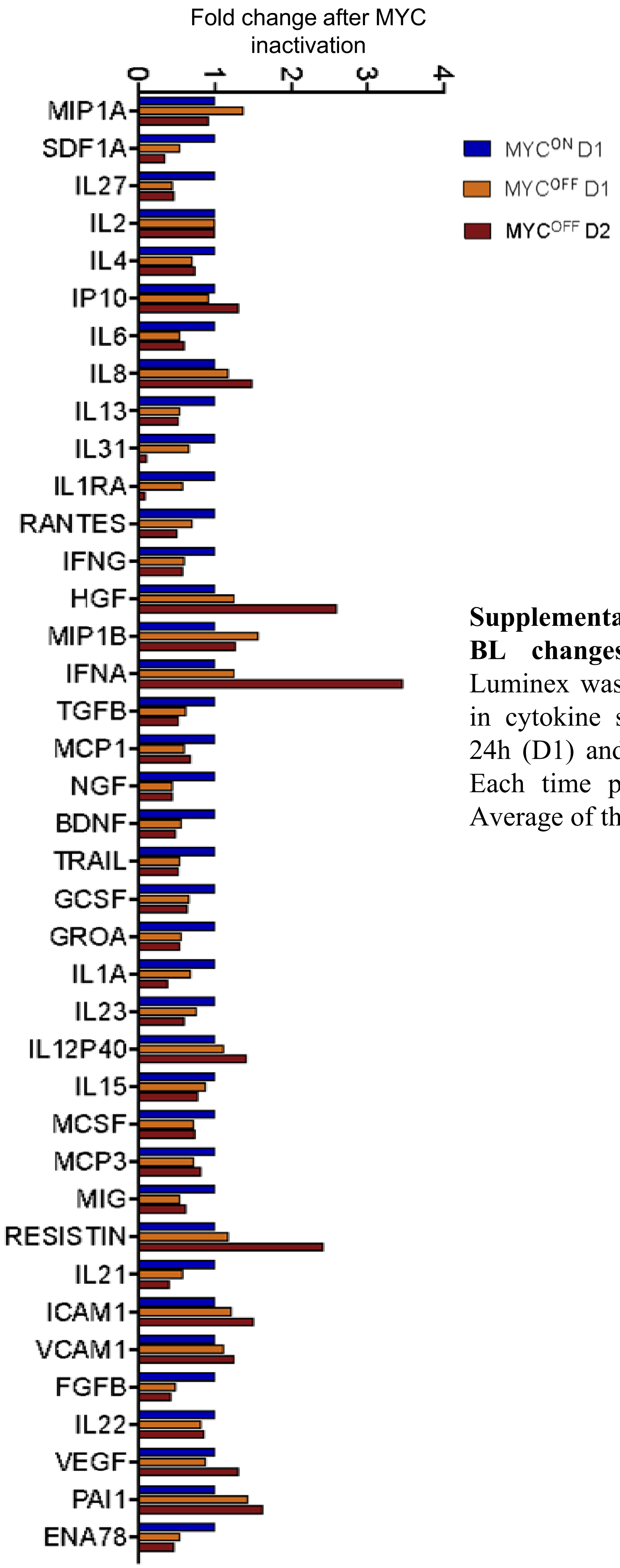

Supplementary Figure 20: MYC inactivation in BL changes the secreted cytokine profile. Luminex was used to evaluate the global changes in cytokine secretion post MYC inactivation for 24h (D1) and 48h (D2) in the P493-6 BL model. Each time point was carried out in duplicates. Average of these duplicates is shown.

Supplementary Figure 21: *Flow cytometry validation of T-lymphoma cell line derived from an SRα-tTA/Tet-O-MYC mouse*

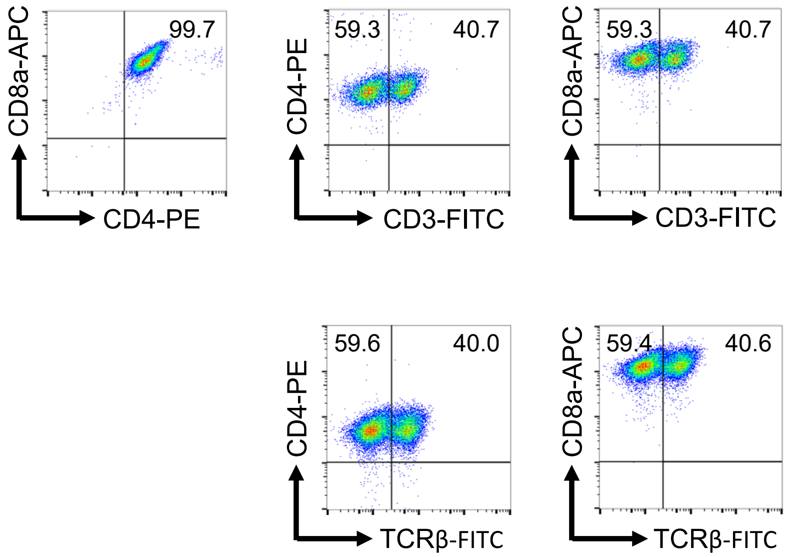

Supplementary Figure 21: Flow cytometry validation of a T-lymphoma cell line derived from an *SRα−tTA/Tet-O-MYC* mouse. Flow cytometry plots depicting the surface expression of T cell markers, such as CD3, CD4, CD8 and TCRβ in a cell line derived from *SRα−tTA /Tet-O-MYC* mice.

Supplementary Figure 22: *Suppression of STAT1/2-Type I IFN signaling in MYC-driven T cell lymphomas*

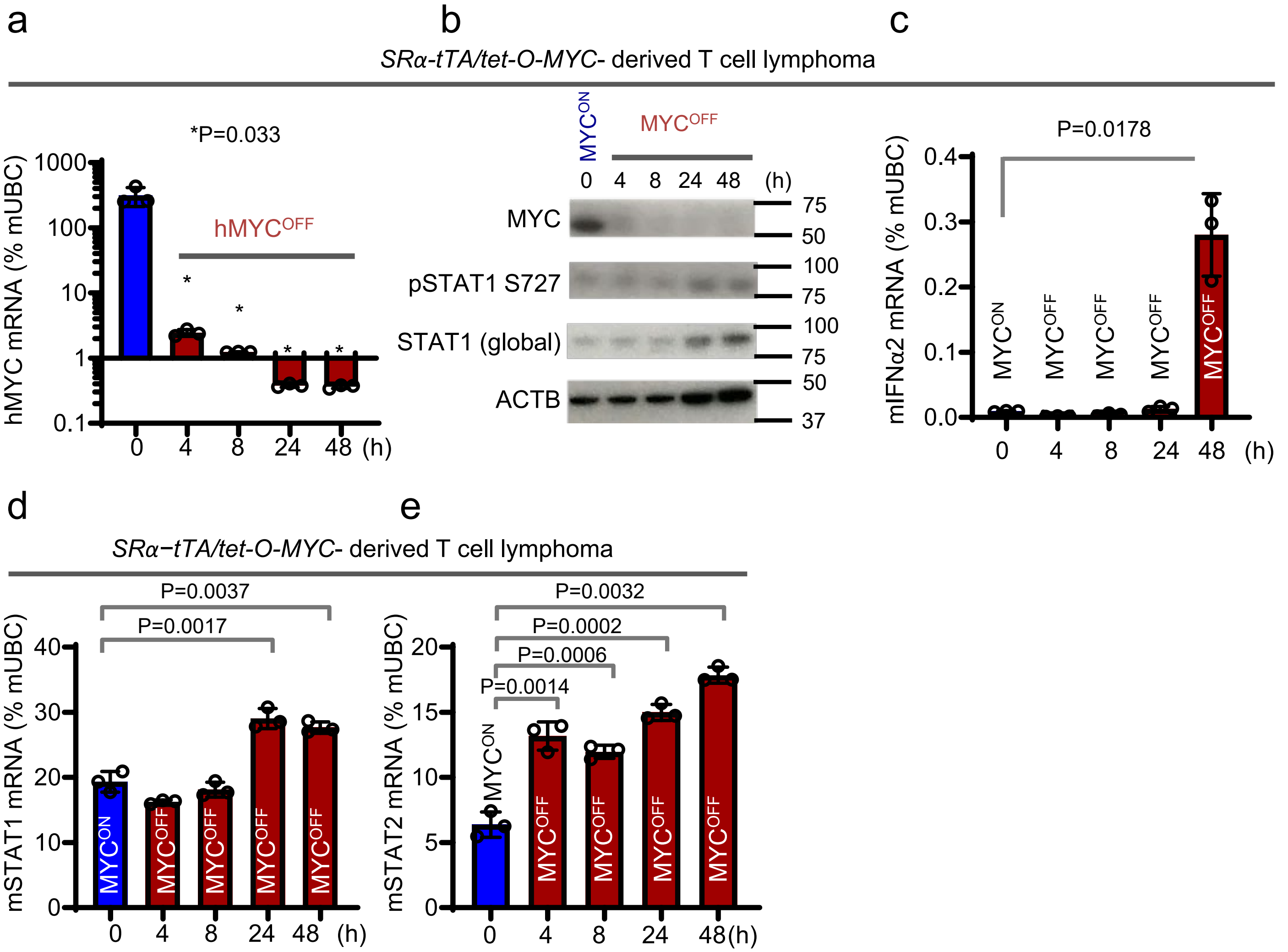

Supplementary Figure 22: Suppression of STAT1/2-Type I IFN signaling in MYC-driven T cell lymphomas. (**a**) Quantitative real time PCR for human MYC transgene in *SRα-tTA MYC* transgenic mouse T-lymphoma cells before and after MYC inhibition by doxycycline for 4, 8, 24 and 48h (n = 3, mean ± s.d). (**b**) Immunoblotting to measure changes in STAT1 activation pre- and post-MYC transgene inactivation in *SRα-tTA* MYC mouse T-lymphoma cells. (**c-e**) Quantitative real time PCR for mType I IFNα2 (c), mSTAT1 (d) and, mSTAT2 (e) in a T-lymphoma cell line derived from an *SRα-tTA/tet-O-MYC* mouse before and after MYC inactivation by doxycycline for 4, 8, 24 and 48 hours (n = 3, mean ± s.d). Two-sided p values have been calculated using the Student's t-test.

Supplementary Figure 23: *Full scan of blot shown in Supplementary Figure 22b*

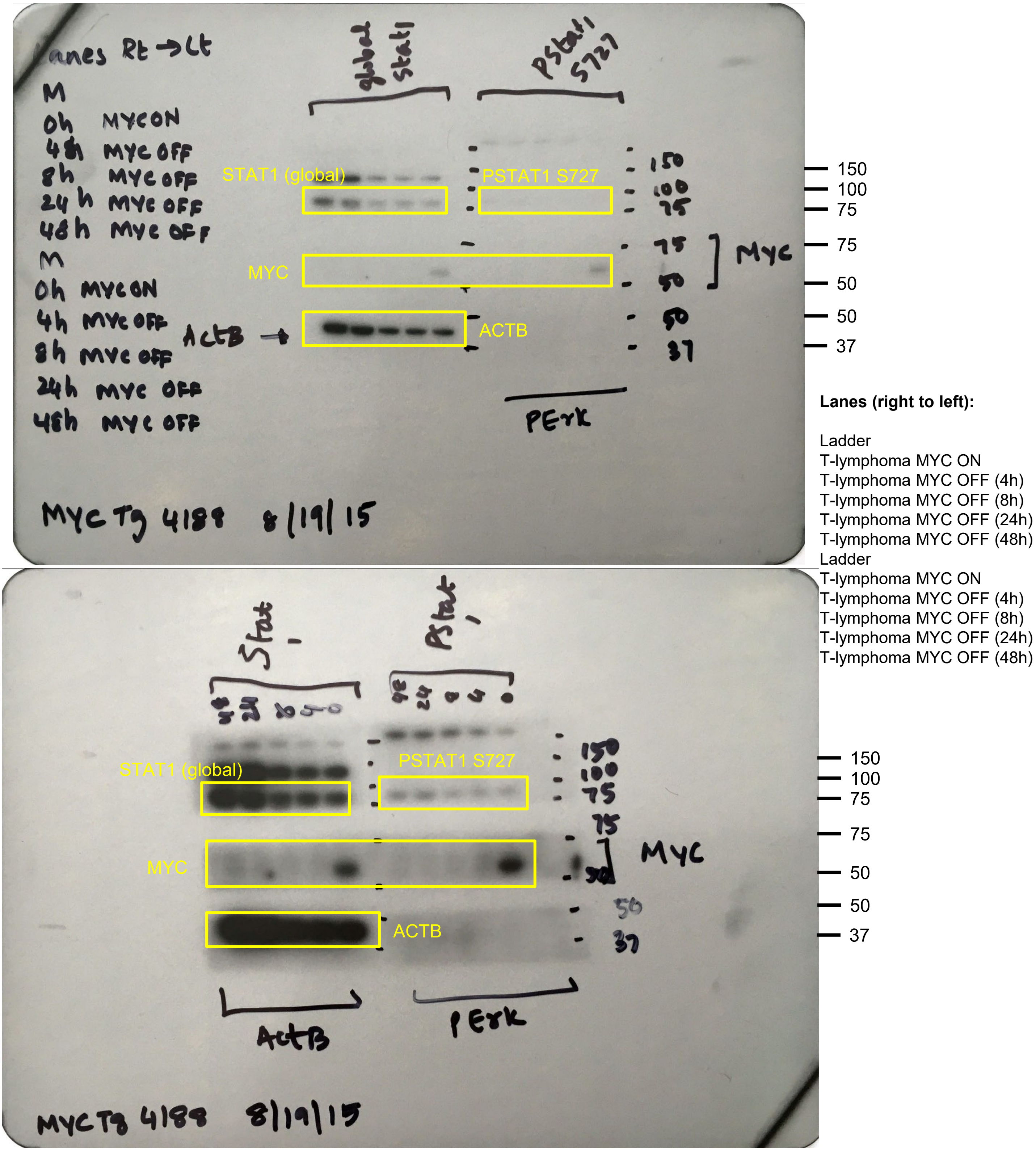

Supplementary Figure 23: Full scan of blot shown in Supplementary Figure 22b. Blot was developed on a film.

Supplementary Figure 24: *MYC binds to STAT1 promoter in MYC-driven lymphomas*

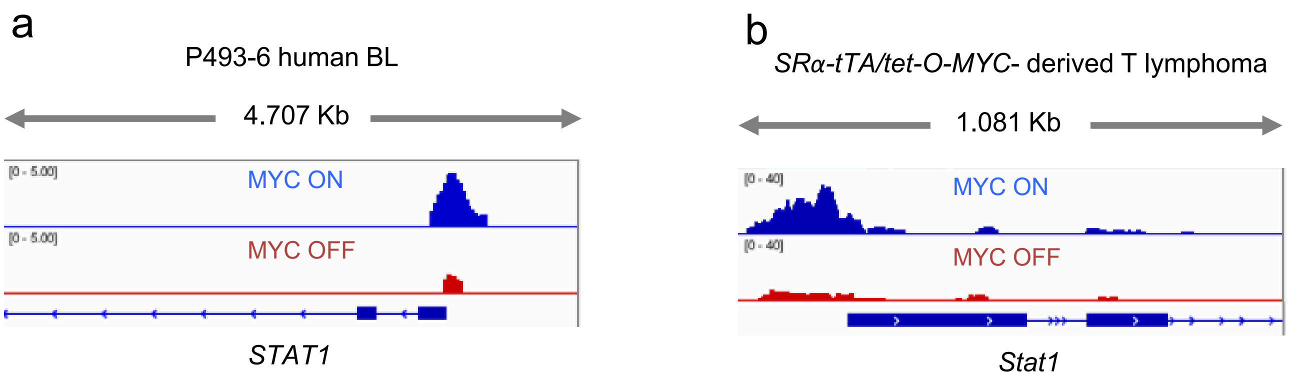

Supplementary Figure 24: MYC binds to STAT1 promoter in MYC-driven lymphomas. (a-b) ChIP sequencing depicting binding of MYC to STAT1 promoter in P493-6 (a) and *SRα-tTA MYC* T-lymphoma (b) cell lines before and after MYC inactivation.

Supplementary Figure 25: *Full scan of blot shown in Figure 5g*

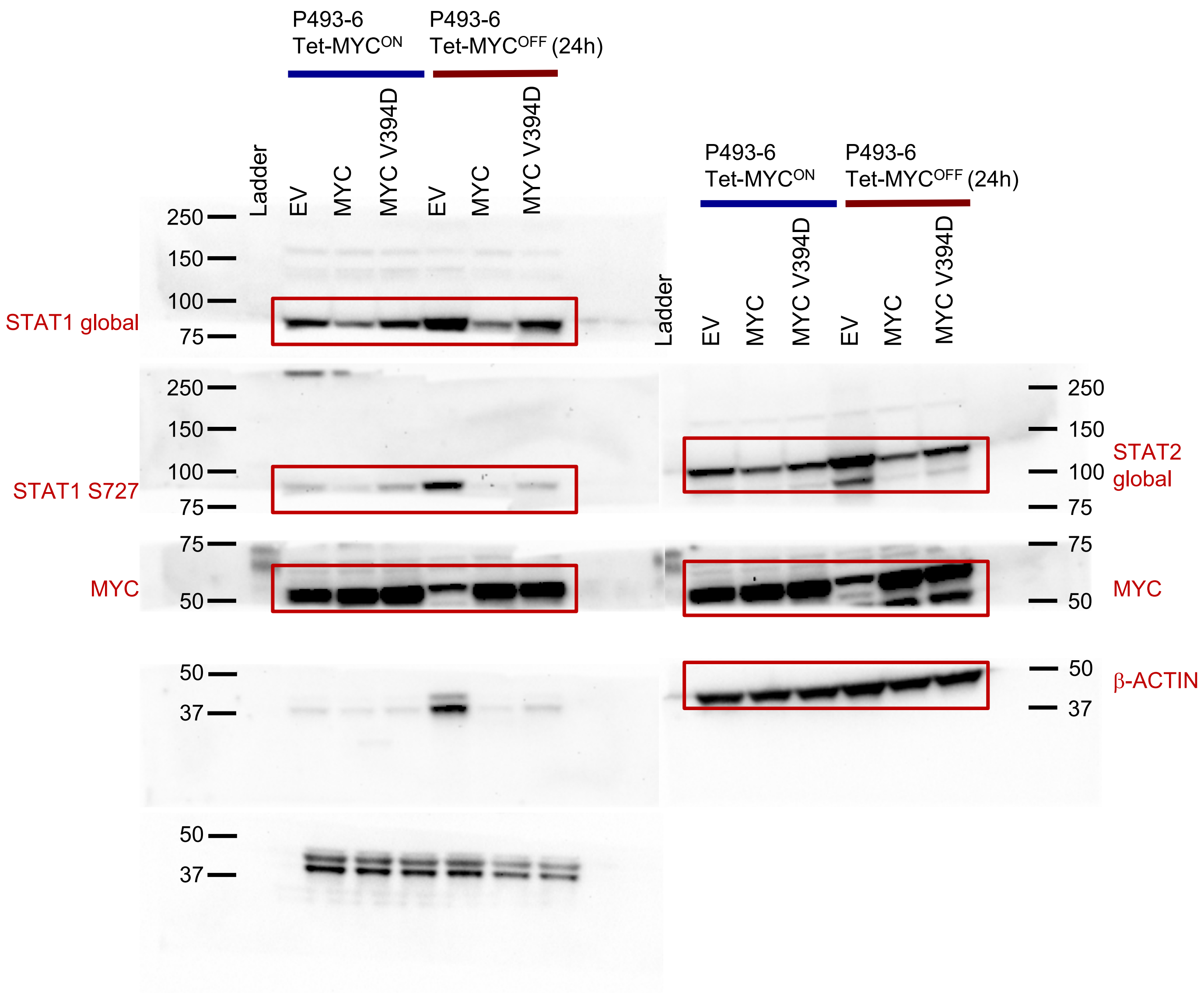

Supplementary Figure 25: Full scan of blot shown in Figure 5g. Blot was developed in Bio-Rad Chemidoc MP Imaging System.

Supplementary Figure 26: *Oncogenic MYC transcriptionally represses STAT1/2-Type I IFN signaling*

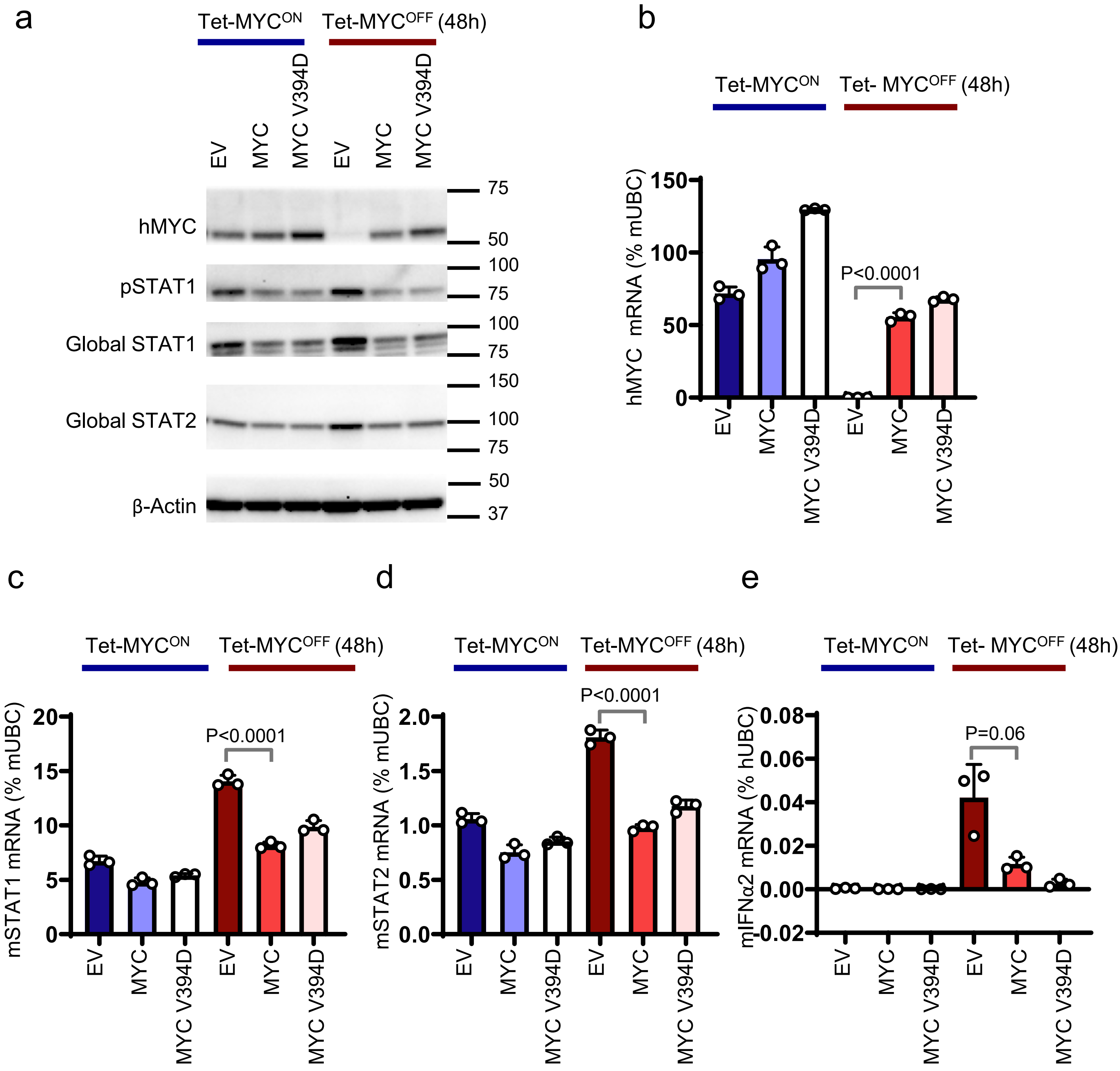

Supplementary Figure 26: Oncogenic MYC transcriptionally represses STAT1/2-Type I IFN signaling. (**a**) Immunoblotting in MYC-driven *SRα-tTA* T-lymphoma cell lines expressing empty vector (EV), MYC overexpression vector (MYC), or a MYC mutant that fails to bind MIZ1 (MYC V394D) before (*Tg-MYC^{ON}*) and after (*Tg-MYC^{OFF}*) MYC inactivation. **(b-e)** Quantitative real time PCR for hMYC (b), mSTAT1 (c), mSTAT2 (d), and mIFNα2 (e) in MYC-driven *SRα-tTA* T-lymphoma cell lines expressing empty vector (EV), MYC overexpression vector (MYC), or a MYC mutant that fails to bind MIZ1 (MYC V394D) before (*Tg-MYC^{ON}*) and after (*Tg-MYC^{OFF}*) MYC inactivation (n = 3, mean ± s.d). Two-sided p values have been calculated using the Student's t-test.

Supplementary Figure 27: *Full scan of blot shown in Supplementary Figure 26a*

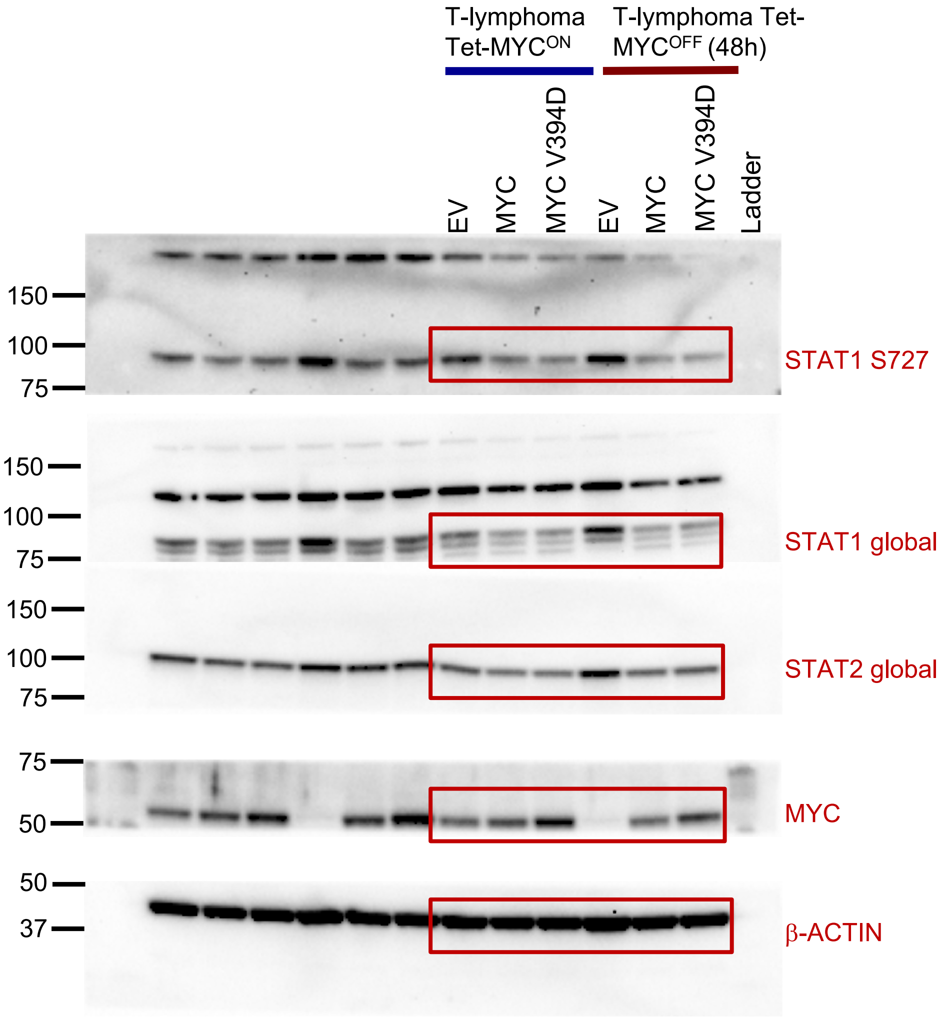

Supplementary Figure 27: Full scan of blot shown in Supplementary Figure 26a. Blot was developed in Bio-Rad Chemidoc MP Imaging System.

Supplementary Figure 28: *Depletion of NK cells increases aggressiveness of MYC-driven T-lymphomas*

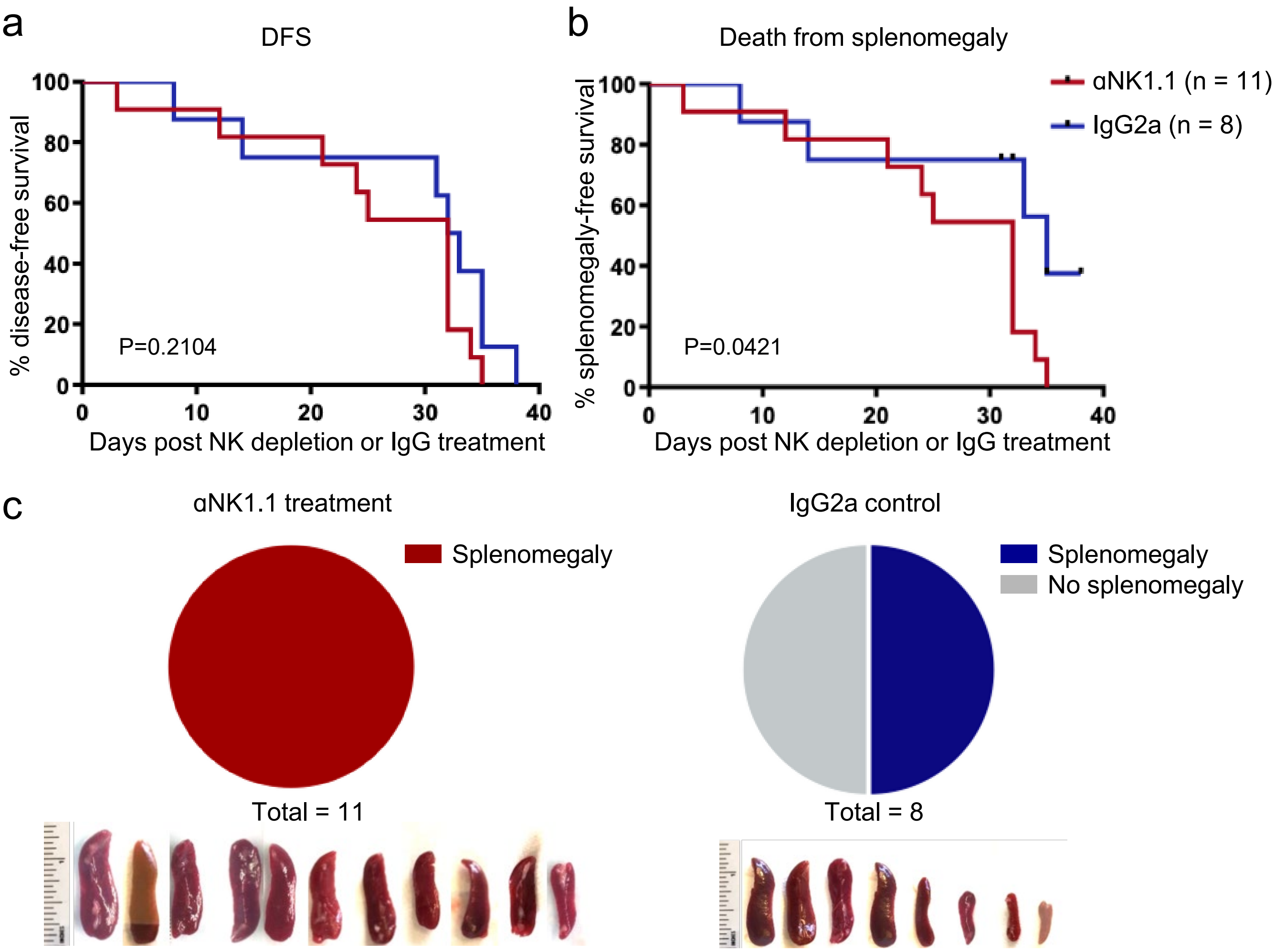

Supplementary Figure 28: Depletion of NK cells increases aggressiveness of MYC-driven T-lymphomas. Comparison of survival probabilities of lymphomagenic *SRα-tTA/tet-O-MYC* mice that were treated with either control IgG2a (n = 8) or NK1.1 antibody (n = 11) to deplete NK cells before overt lymphoma manifestation. (**a**) Disease-free survival (DFS). (**b**) Death from splenomegaly. P-value has been calculated using the log-rank test. (**c**) Pie charts and pictures indicating splenomegaly of the *SRα-tTA/tet-O-MYC* mice that were treated with either control IgG2a or NK1.1 depleting antibody antibody. P-values were calculated by log-rank test.

Supplementary Figure 29: *Isolation and purification of syngeneic NK cells for adoptive transfer into MYC-driven T-lymphoma bearing NSG recipients*

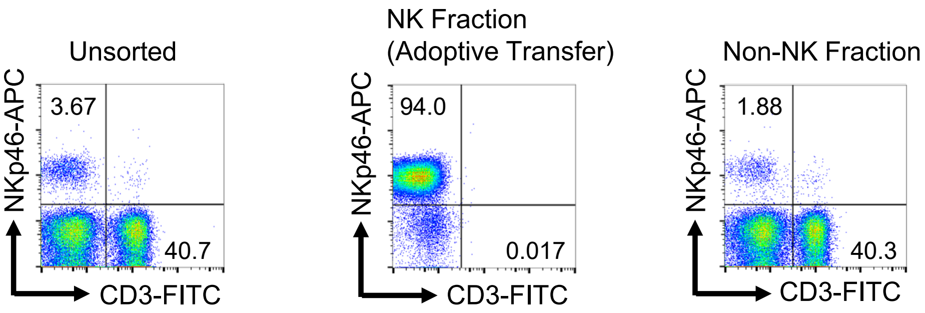

Supplementary Figure 29: Isolation and purification of syngeneic NK cells for adoptive transfer into MYC-driven T-lymphoma bearing NSG recipients. Validation of purity of NK cells isolated by magnetic activated cell sorting (MACS) by flow cytometry for surface CD3, NKp46 and CD49b.

Supplementary Figure 30: *Oncogenic MYC suppresses NK cell-mediated immune surveillance of lymphoid malignancies.*

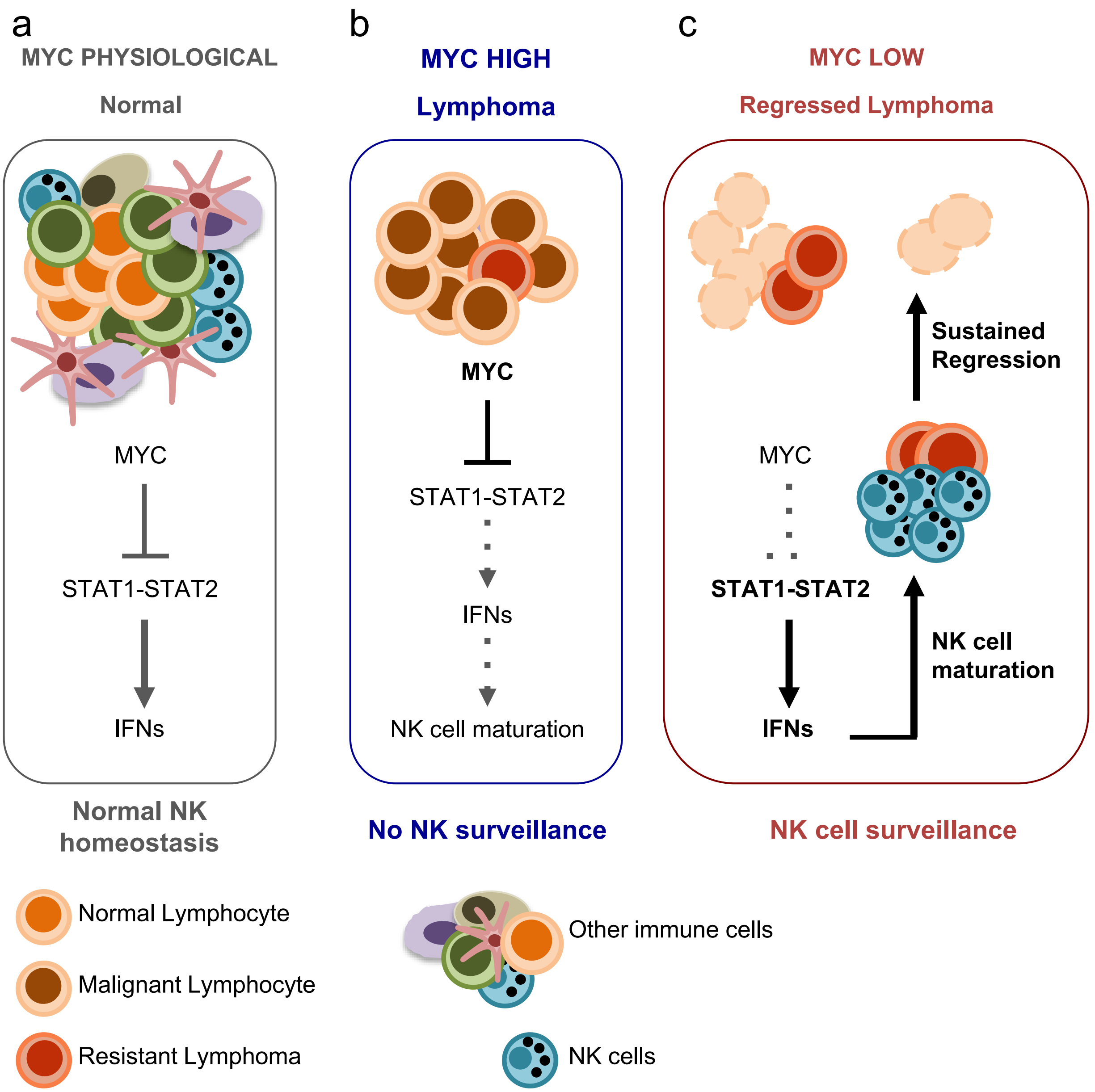

Supplementary Figure 30: Oncogenic MYC suppresses NK cell-mediated immune surveillance of lymphoid malignancies. (**a**) Normal distribution of splenic immune compartments in the absence of lymphoma. (**b**) MYC-induced lymphomagenesis is associated with the modulation of cytokines and immune subsets in the tumor microenvironment. Activation of oncogenic MYC reduces the secretion of anti-lymphomagenic cytokines, such as Type I IFNs. The altered cytokine milieu excludes NK cells reducing immune surveillance of the lymphoma. (**c**) MYC inactivation triggers Type I IFN secretion by the lymphoma and NK-mediated immune surveillance. Hyperactivated signaling arms under each condition are depicted in bold black.

Supplementary Figure 31: *Gating strategy for CyTOF and flow cytometry*

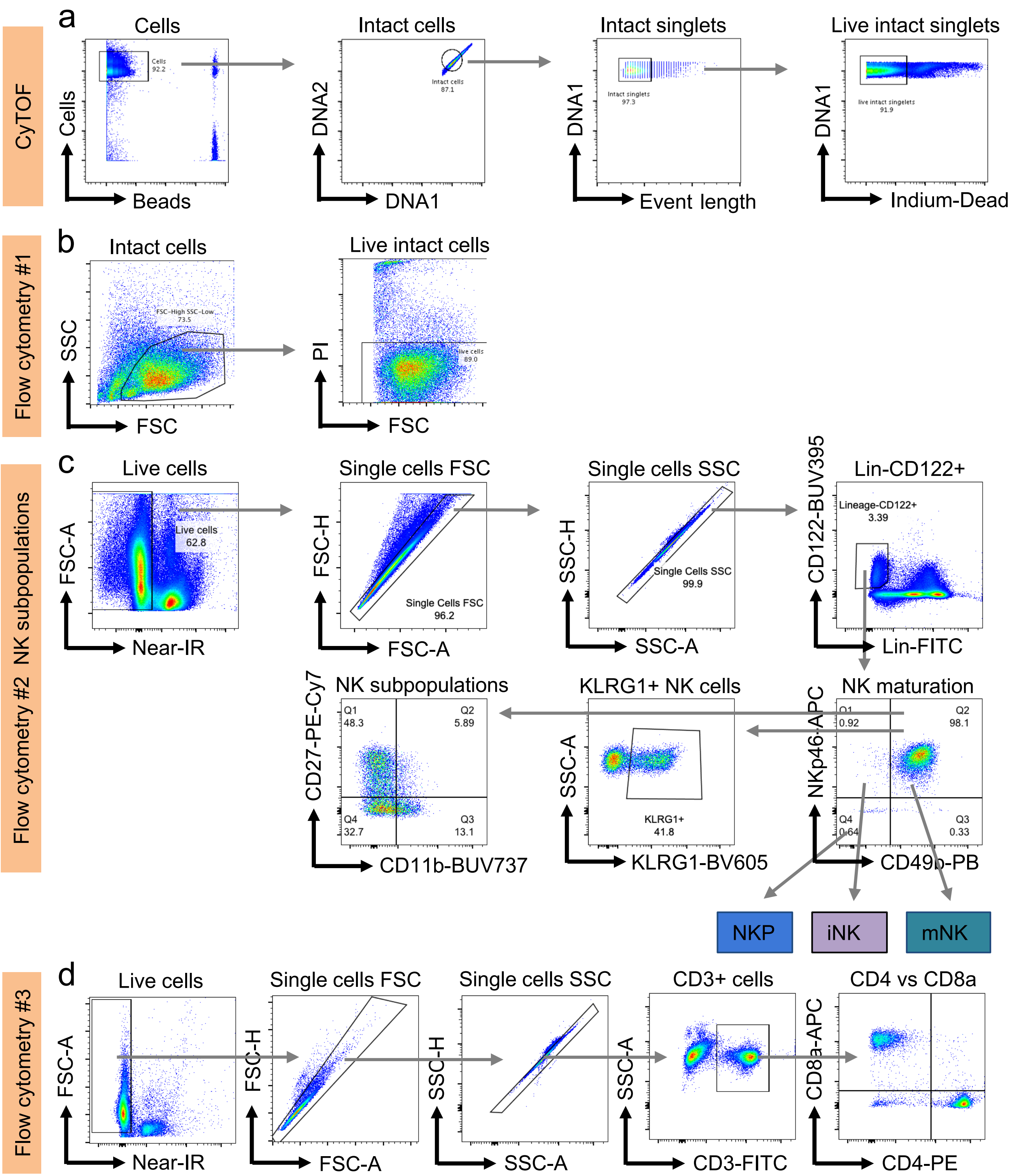

Supplementary Figure 31: Gating strategy for CyTOF and flow cytometry. (a) Dot-plot showing the gating strategy for CyTOF data in Figure 1 and 5b and Supplementary Figure 2, 3, 7, and 8. **(b)** Dot-plots showing the flow cytometry gating strategy used for Figure 2 and Supplementary Figure 5, 15, 20, and 25. **(c)** Dot-plot showing the flow cytometry gating strategy used for analysis of NK maturation in Figure 3a-i, and 4h-i, and Supplementary Figure 9, 10, 12, and 16. **(d)** Dot-plots showing the flow cytometry gating strategy used in Supplementary Figure 11. All flow cytometry gating strategies show a representative spleen sample.

Supplementary Figure 31 contd.: *FMO controls for gating of NK subpopulations*

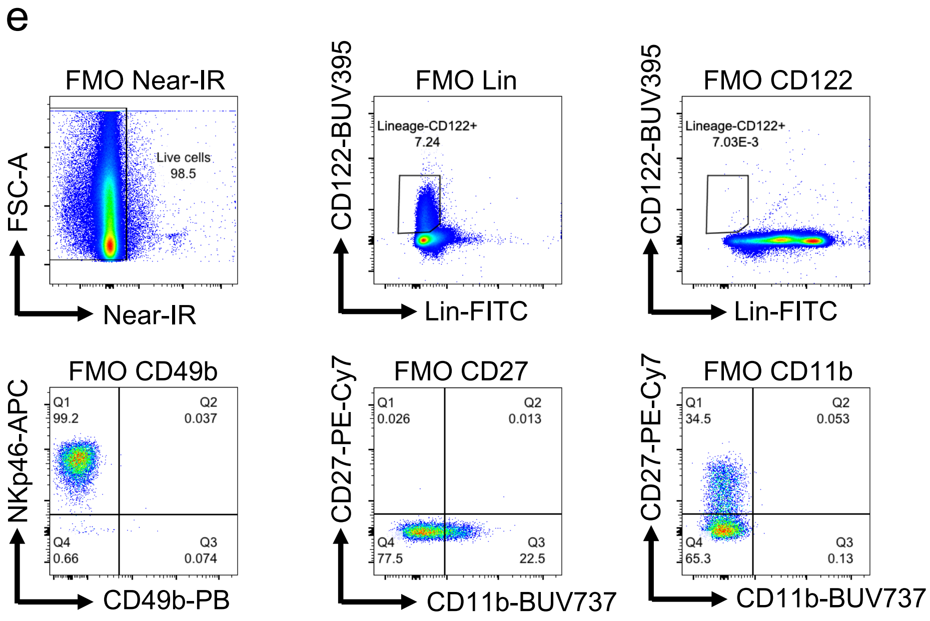

Supplementary Figure 31 contd.: FMO controls for gating of NK subpopulations. Dot-plots showing the fluorescence-minus-one (FMO) control samples used for setting the boundaries between "negative" and "positive" for gating of NK subpopulations.

Supplementary Table 1: *Antibodies used for CyTOF immunophenotyping (mouse)*

Antigen	Metal Tag	Dilution	Source
Live-Dead	In115	1:50	HIMC, Stanford University
Ly6G	Pr141	1:50	Fluidigm Corporation
CD11c	Nd142	1:50	Fluidigm Corporation
CD11b	Nd143	1:50	Fluidigm Corporation
Ly6C	Dy162	1:50	Fluidigm Corporation
CD3	Sm152	1:50	Fluidigm Corporation
CD8a	Er168	1:50	Fluidigm Corporation
CD4	Nd145	1:50	Fluidigm Corporation
TCRγδ	Tb159	1:50	Fluidigm Corporation
TCRβ	Tm169	1:50	Fluidigm Corporation
CD19	Sm149	1:50	Fluidigm Corporation
MHCII	Yb174	1:50	Fluidigm Corporation
NKp46	Eu153	1:50	Fluidigm Corporation
CD49b	Er170	1:50	Fluidigm Corporation
Ter119	Sm154	1:50	Fluidigm Corporation

Supplementary Table 2: *Antibodies used for Phospho-CyTOF (human)*

Antigen	Metal Tag	Dilution	Source
Live-Dead	In115	1:50	HIMC, Stanford
Ly6G	Pr141	1:50	HIMC, Stanford
CD19	Nd142	1:50	HIMC, Stanford
CD117	Nd143	1:50	HIMC, Stanford
CD11b	Nd144	1:50	HIMC, Stanford
CD4	Nd145	1:50	HIMC, Stanford
CD20	Sm147	1:50	HIMC, Stanford
CD7/ CD45RO	Sm149	1:50	HIMC, Stanford
CD123	Eu151	1:50	HIMC, Stanford
CD27	Sm152	1:50	HIMC, Stanford
CD45RA	Eu153	1:50	HIMC, Stanford
CD45	Sm154	1:50	HIMC, Stanford
pP38	Gd156	1:50	HIMC, Stanford
CD24	Gd157	1:50	HIMC, Stanford
pSTAT3	Gd158	1:50	HIMC, Stanford
CD11c	Tb159	1:50	HIMC, Stanford
CD14	Gd160	1:50	HIMC, Stanford
IgD	Dy161	1:50	HIMC, Stanford
pERK1/2	Dy162	1:50	HIMC, Stanford
IκBtot	Dy163	1:50	HIMC, Stanford
CD25	Dy164	1:50	HIMC, Stanford
pS6	Ho165	1:50	HIMC, Stanford
CD16	Er166	1:50	HIMC, Stanford
CD38	Er167	1:50	HIMC, Stanford
CD8	Er168	1:50	HIMC, Stanford
pSTAT1	Tm169	1:50	HIMC, Stanford
CD3	Er170	1:50	HIMC, Stanford
pSTAT5	Yb172	1:50	HIMC, Stanford
pPLCγ2	Yb173	1:50	HIMC, Stanford
HLADR	Yb174	1:50	HIMC, Stanford
CD56	Lu175	1:50	HIMC, Stanford
CD127	Yb176	1:50	HIMC, Stanford

Supplementary Table 3: *Antibodies used for flow cytometry*

Surface Antigen	Fluorophore	Clone ID	Dilution	Source
CD3ε	FITC	145-2C11	1:200	BD Pharmingen
CD4	PE	RM4-5	1:100	BD Pharmingen
CD8α	APC	53-6.7	1:200	BD Pharmingen
CD11b	BUV737	M1/70	1:300	BD Optibuild
CD19	FITC	1D3/CD19	1:500	BioLegend
CD27	PE-Cy7	LG.3A10	1:500	BioLegend
CD49b	Pacific Blue	DX5	1:200	BioLegend
CD122	BUV395	TM-B1	1:100	BD Optibuild
CD335 (NKp46)	APC	29A1.4	1:100	BioLegend
KLRG1	Brilliant Violet 605	2F1/KLRG1	1:100	BioLegend
NKG2D	PE	1D11	1:200	BD Pharmingen
TCR-β	FITC	H57-597	1:200	eBioscience
CD16/CD32 (Fc block)	-	2.4G2	1:50	BD Pharmingen
Live/dead	Near-IR	-	1:10,000	Thermo Fisher

Supplementary Table 4: *Antibodies used for immunoblotting*

Antigen	Clone ID	Dilution	Source
MYC	N-262	1:1000	Santa Cruz Biotechnology
ACTB	ab8227	1:10,000	Abcam
STAT1	9172	1:1000	Cell Signaling Technology
Phospho S727 STAT1	9177	1:1000	Cell Signaling Technology
STAT2	D9J7L	1:1000	Cell Signaling Technology

Supplementary Table 5: *Lentiviral and retroviral vectors used in the study*

Vector	Type	Source
pMSCV Luciferase IRES-puro	Retroviral	Addgene
pRRL	Lentiviral	Lab of Martin Eilers
pRRL hMYC	Lentiviral	Lab of Martin Eilers
pRRL hMYC V394D	Lentiviral	Lab of Martin Eilers

Transfections of the above retroviral constructs were performed as discussed in the online methods section.

Supplementary Table 6: *Sequences of oligonucleotide primers used for quantitative PCR*

Mouse primers

mIFNA2_F	5'-AAGGTCCTGGCACAGATGAG-3'
mIFNA2_R	5'-GGAGGGTTGTATTCCAAGCA-3'
mSTAT1_F	5'-TGGTGAAATTGCAAGAGCTG-3'
mSTAT1_R	5'-CAGACTTCCGTTGGTGGATT-3'
mSTAT2_F	5'-GCCTCCATTCTCTGGTTCAA-3'
mSTAT2_R	5'-TCCTCCATCTTGCAGCTCTT-3'
mUBC_F	5'-AGCCCAGTGTTACCACCAAG-3'
mUBC_R	5'-ACCCAAGAACAAGCACAAGG-3'

Human primers

hIFNA2_F	5'-GCAAGTCAAGCTGCTCTGTG-3'
hIFNA2_R	5'-CAAACTCCTCCTGGGGAAAT-3'
hSTAT1_F	5'-CCGTTTTCATGACCTCCTGT-3'
hSTAT1_R	5'-TGAATATTCCCCGACTGAGC-3'
hSTAT2_F	5'-GGAACAGCTGGAGACATGGT-3'
hSTAT2_R	5'-GGCTGGGTTTCTACCACAAA-3'
hMYC_F	5'-CTGCGACGAGGAGGAGAACT-3'
hMYC_R	5'-GGCAGCAGCTCGAATTTCTT-3'
hUBC_F	5'-CTGGAAGATGGTCGTACCCTG-3'
hUBC_R	5'-GGTCTTGCCAGTGAGTGTCT-3'
